# Neural Network Evidence for the Coupling of Presaccadic Visual Remapping to Predictive Eye Position Updating

**DOI:** 10.3389/fncom.2016.00052

**Published:** 2016-06-02

**Authors:** Hrishikesh M. Rao, Juan San Juan, Fred Y. Shen, Jennifer E. Villa, Kimia S. Rafie, Marc A. Sommer

**Affiliations:** ^1^Department of Biomedical Engineering, Pratt School of Engineering, Duke UniversityDurham, NC, USA; ^2^Department of Neurobiology, Duke School of Medicine, Duke UniversityDurham, NC, USA; ^3^Center for Cognitive Neuroscience, Duke UniversityDurham, NC, USA

**Keywords:** visual stability, eye movements, saccades, Topographica, recurrent networks

## Abstract

As we look around a scene, we perceive it as continuous and stable even though each saccadic eye movement changes the visual input to the retinas. How the brain achieves this perceptual stabilization is unknown, but a major hypothesis is that it relies on presaccadic remapping, a process in which neurons shift their visual sensitivity to a new location in the scene just before each saccade. This hypothesis is difficult to test *in vivo* because complete, selective inactivation of remapping is currently intractable. We tested it *in silico* with a hierarchical, sheet-based neural network model of the visual and oculomotor system. The model generated saccadic commands to move a video camera abruptly. Visual input from the camera and internal copies of the saccadic movement commands, or corollary discharge, converged at a map-level simulation of the frontal eye field (FEF), a primate brain area known to receive such inputs. FEF output was combined with eye position signals to yield a suitable coordinate frame for guiding arm movements of a robot. Our operational definition of perceptual stability was “useful stability,” quantified as continuously accurate pointing to a visual object despite camera saccades. During training, the emergence of useful stability was correlated tightly with the emergence of presaccadic remapping in the FEF. Remapping depended on corollary discharge but its timing was synchronized to the updating of eye position. When coupled to predictive eye position signals, remapping served to stabilize the target representation for continuously accurate pointing. Graded inactivations of pathways in the model replicated, and helped to interpret, previous *in vivo* experiments. The results support the hypothesis that visual stability requires presaccadic remapping, provide explanations for the function and timing of remapping, and offer testable hypotheses for *in vivo* studies. We conclude that remapping allows for seamless coordinate frame transformations and quick actions despite visual afferent lags. With visual remapping in place for behavior, it may be exploited for perceptual continuity.

## Introduction

Frequent eye movements known as saccades allow us look around a visual scene rapidly, but at the cost of disrupting the continuity of visual information. How the brain uses the “jumpy” input from the retinas to construct a continuous, stable percept is a long-standing question in systems neuroscience (Sperry, 1950; Holst and Mittelstaedt, 1971; von Helmholtz, 2000). The phenomenon of presaccadic visual remapping, discovered by Goldberg and colleagues (Duhamel et al., 1992), is considered a likely mechanism for contributing to perceptual stability (Sommer and Wurtz, 2008; Wurtz, 2008; Wurtz et al., 2011; Cavanaugh et al., 2016). Neurons that remap use predictive oculomotor information to shift their locus of visual analysis just before each saccade (Walker et al., 1995; Umeno and Goldberg, 1997; Nakamura and Colby, 2002). In cerebral cortical areas such as the frontal eye field (FEF) and parietal and occipital regions, a substantial component of this shift is parallel to the saccade (Sommer and Wurtz, 2006), although the full dynamics of the shifts are not yet settled (Tolias et al., 2001; Sommer and Wurtz, 2004a,b; Zirnsak et al., 2014; Mayo et al., 2015, 2016; Neupane et al., 2016, in press for review see Marino and Mazer, 2016). The effect of remapping a visual response parallel to the saccade is to sample the location of visual space, the “future field,” that will be occupied by the classical receptive field after the saccade. This provides the opportunity for distinguishing changes in visual input that arise from self-motion from those due to external image movement, and some neurons in FEF make this distinction (Crapse and Sommer, 2012). Hence, although we have a long way to go in understanding subjective visual continuity across saccades, the prevailing hypothesis is that it is attributable to presaccadic visual remapping (Melcher and Colby, 2008).

The physiological basis of presaccadic visual remapping has been elucidated most fully in the FEF. First, a pathway in the rhesus monkey brain was identified that carries information about impending saccades to the FEF (Lynch et al., 1994; Sommer and Wurtz, 2002). This pathway arises from the superior colliculus (SC), which encodes saccades as vectors relative to the fovea (Fuchs et al., 1985; Sparks, 1986; Moschovakis, 1996). Signals from the SC are relayed through the lateral edge of the mediodorsal nucleus of the thalamus (Sommer and Wurtz, 2004a). Both of the SCs, left and right, appear to send convergent signals to each FEF, providing individual FEF neurons with information about all vectors of saccades (Crapse and Sommer, 2009). Second, it is known that the ascending saccadic vector signals, or “corollary discharge,” contribute to presaccadic visual remapping in FEF. When the pathway from SC through MD thalamus is inactivated in monkeys, remapping in FEF is disrupted (Sommer and Wurtz, 2004b, 2006) and stable visual perception is impaired (Cavanaugh et al., 2016). Similar deficits in visual stability result from damage to analogous thalamic areas in monkeys and humans (Bellebaum et al., 2005; Ostendorf et al., 2010; Cavanaugh et al., 2016). Third, we are starting to understand remapping at the microcircuit level within FEF. The remapping is relatively fragmented in thalamic-recipient FEF layer IV, with saccade-related modulations of classical receptive field and future field distributed across separate putative neuron types, but is integrated into more cohesive remapping at FEF output layer V (Shin and Sommer, 2012).

In addition to corollary discharge from the SC, which represents the vector of the next saccade (Sommer and Wurtz, 2006), information about the upcoming static eye position is available. Such “predictive eye position” signals are found in the thalamus (Schlag-Rey and Schlag, 1984; Wyder et al., 2003; Tanaka, 2007) and brainstem (Lopez-Barneo et al., 1982; Fukushima, 1987; Crawford et al., 1991), and they seem to influence activity in the cerebral cortex (Schlag et al., 1992; Boussaoud et al., 1993, 1998; Wang et al., 2007). The pathways that carry predictive eye position signals to cerebral cortex are still unknown, however, limiting our ability to test their influence on presaccadic remapping.

Therefore, much has been learned about the mechanics of presaccadic remapping, especially in the FEF. Yet a key question—the relation between presaccadic remapping and perceptual visual stability—remains unanswered. To a large extent we have reached the limits of what can be revealed neurophysiologically in the animal model of choice, the behaving rhesus monkey. To conclusively demonstrate the link between presaccadic remapping and perceptual stability, we would need to test behavioral indicators of a monkey's perceptual stability while selectively inactivating all neurons that remap while leaving all other visual neurons unaffected. Such an experiment is not yet possible *in vivo*. Here we undertook a new approach: constructing a biologically plausible model of the FEF to gain insights not yet achievable *in vivo* and generate new, testable hypotheses for neural experiments. In contrast to prior models of remapping (Andersen et al., 1993; White and Snyder, 2004; Deneve et al., 2007; Keith and Crawford, 2008), we use a less abstract, more neuromorphic architecture that simplifies physiological interpretation. Our results reveal a previously unappreciated synchronization between remapping and predictive eye position signals. This temporal coupling explains several prior findings, is amenable to laboratory validation or refutation, and provides a novel conceptual framework for understanding how remapping contributes to visual stability.

## Materials and methods

### Overview

We used a map-based neural network system developed originally for modeling topographically organized “layers” or sheets of visually-sensitive neurons in occipital cortex (primary visual area V1; Bednar and Miikkulainen, 2003; Bednar et al., 2004; Bednar, 2008). In our model, sheets of the neurons formed a simulated retina and the early dorsal visual system on the sensory side, and a simulated SC and thalamus on the oculomotor side. The visual input to the model was provided by a video camera, and the oculomotor output was provided by the SC along two pathways: a “motor” branch that controlled the camera to move it with simulated saccades, and a “corollary discharge” branch that provided the model with copies of the saccadic commands. The visual and corollary discharge streams of information converged in a sheet that simulated the FEF. The output from the FEF was combined with eye position signals to yield a reference frame transformation appropriate for controlling arm movements of a real or simulated humanoid robot that pointed to objects in its workspace. Embodied in this system, the model had to learn to guide accurate reaches despite camera saccades.

Such a computational model has advantages but also challenges. For our study, the main problem is that, of course, a model or robot does not experience “perceptual” stability. But this is similar to performing experiments with a monkey. We can ask neither entity what they experience, and should not presume that they “experience” anything. Even when studying visual perception in humans, conclusions depend on objective measurements more than subjective reports. In all such experiments, one needs to establish an operational definition of visual stability. We tested whether the model achieved “useful” visual stability, operationally defined as continuously accurate localization of a visual object (measured by robotic pointing) despite saccadic movements of visual input (caused by moving the camera). Our rationale was that useful visual stability is the objective, observable counterpart to the subjective, perceptual visual stability. Moreover, for the purpose of navigating through and interacting with the world—the primary goal of all animals—useful visual stability is what matters.

Using this experimental paradigm, we tested whether useful visual stability in our system depended on presaccadic remapping in the modeled FEF. A positive result would support the hypothetical link between remapping and visual stability. A negative result would demonstrate that presaccadic remapping is not necessary for useful visual stability, which in turn would weaken its putative association with perceptual stability. After training the model, we “lesioned” different signals within it—specifically, the visual input and the corollary discharge pathways—to assess their contributions to presaccadic remapping. The results yielded comparisons with prior *in vivo* inactivation data and predictions for future *in vivo* experiments.

### The model

We used a software package called Topographica (http://www.topographica.org), designed originally for modeling visual cortex (Bednar, 2009), to create large scale, hierarchical, connected neural network maps. In Topographica, the fundamental unit is a sheet of neurons, rather than a neuron or part of a neuron. Sheets can be interconnected as a function of receptive field location so as to preserve topographic relationships in visual pathways. The advantage of abstracting away the details of intracellular dynamics was that we could focus on the emergent properties within multiple regions of the brain.

**Figure 1:**
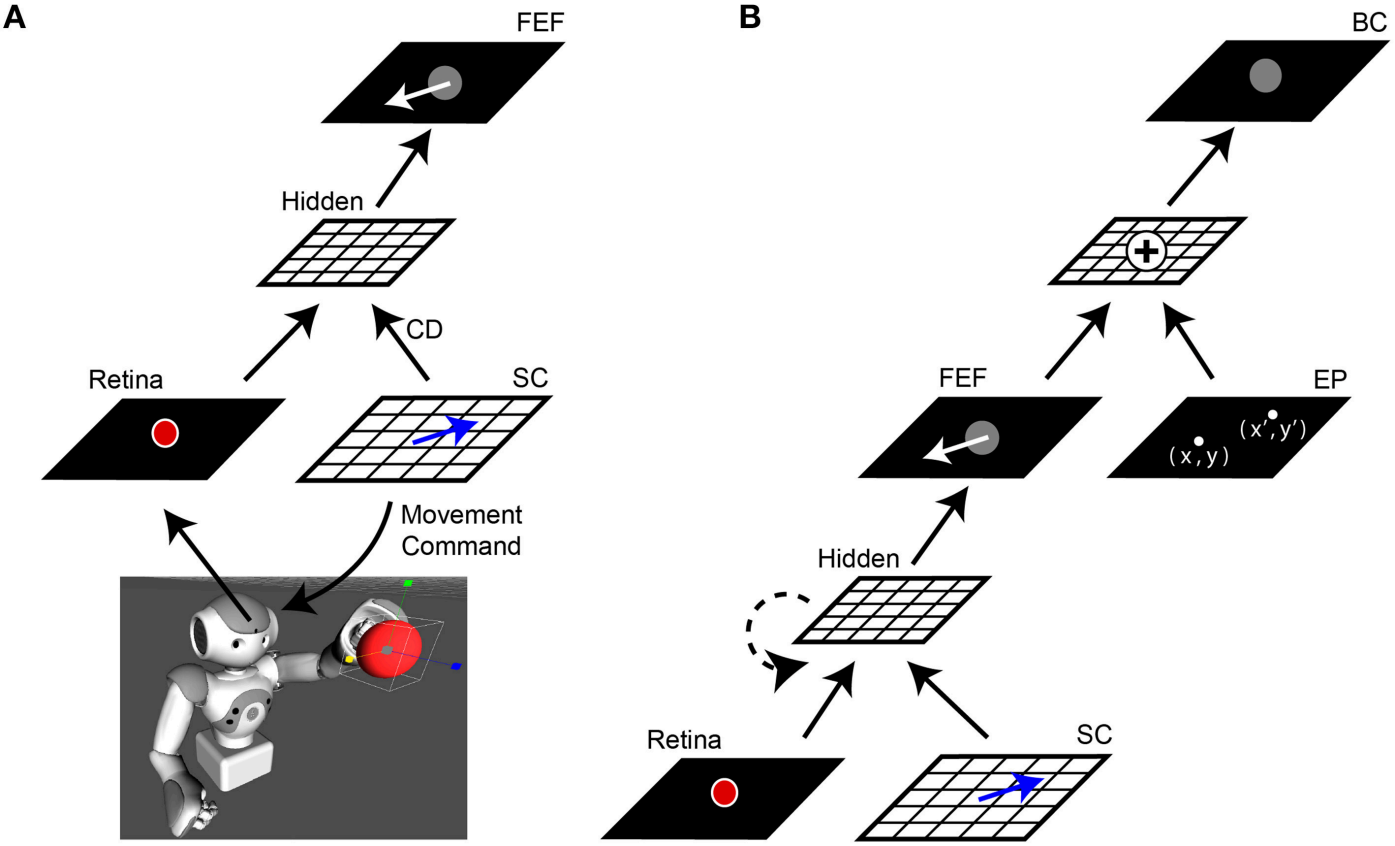
**Network architecture. (A)** Central elements of the neural network “core model” that guided the robot. The camera within the robot's head fed visual information, specifically the image of a red ball, to the Retina layer. Movement commands, generated on the SC layer (blue arrows), controlled rotation of the “eye” (i.e., camera fixed in robot's head). A copy of the SC motor command, the corollary discharge (CD) signal, was combined with visual information in a Hidden layer, the output of which reached the FEF layer. The eye movement displaces (in the opposite direction) the projected location of the red ball on the retina and therefore, after an afferent lag, also on the FEF sheet (white arrow). **(B)** Complete model. The central network in **(A)** was expanded upon by adding a representation of eye position (EP layer) relative to body. The output of the FEF layer was combined with EP signals to yield a Body-centered spatial representation of the object (BC layer). The output of BC guided the robot's arm to the ball. In some experiments, a recurrent connection was added to the lower hidden layer (dashed line). In all experiments, the training was guided only by the final “behavioral” error in pointing to the ball, with all internal sheets changing to optimize performance of the system as a whole.

We will describe the central architecture of the model first (Figure 1A). Visual information from the robot's camera served as input to the network (30 Hz, 1288 × 968 pixels, field of view 61° horizontally × 47° vertically). This camera served as the “eye” for an Aldebaran NAO robot (https://www.aldebaran.com/en/cool-robots/nao). The camera image projected the scene of the robot's workspace onto the model's *Retina* layer. The experiments we report here used the Aldebaran robot simulation software to generate virtual workspace objects and reaches, to simplify the experiment and avoid physical confounds (e.g., potential changes in the robot arm from overuse and imprecise locations or vibrations of reach targets), but all results were the same in experiments that used real robots, objects, and reaches. In simulations, the virtual robot was stripped of all non-model visual guidance and all internal state cues that could assist reaching performance, such as feedback about arm position in time. The robot (or its simulation) was presented with a single object, a red ball. From the Retina layer, the image was processed through “dorsal stream” sheets that ignored features such as color or shape but retained object location in retinal coordinates. A motor sheet (*SC* layer) controlled the movements of the camera to simulate saccadic changes in the visual scene (400°/s). Since the camera was affixed within the robot's head, an SC layer command moved the entire head to displace the image on the Retina layer. Hence the whole head was the “eye” for the purpose of this study, and will be called the eye in the rest of this report. An exact copy of the movement command used to generate each saccade, a simulated corollary discharge, was sent upstream to high-level layers of the network. The visual and saccade vector information converged at a sheet we called the *FEF* layer due to its connectional similarities to the primate brain FEF (for review, see Sommer and Wurtz, 2008).

In the complete model (Figure 1B), a representation of the camera orientation was provided in an Eye Position (*EP*) layer. The outputs from this layer and the FEF layer were combined in a Body-centered (*BC*) layer to guide the arm.

### The task

The robot's task was to point accurately to the ball, regardless of how the camera eye might be moving. Each attempt to point the ball constituted one trial. The general sequence of events during a single trial was as follows (Figure 1B):

1. Visual information from the Retina layer flowed through the network to the FEF layer.
2. The robot pointed to the ball, based on the output of the BC layer. Initially, EP signals are zero, analogous to looking straight ahead. The ball could be anywhere in the workspace as long as it was fully visible on the Retina layer. At every time step, a coordinate is decoded from the BC layer and provided as a target for the robot.
3. Then, a perturbation of the image of the ball might occur. On some trials the ball moved, on other trials the camera moved, and on some trials neither moved. If the camera moved, this was due to activity generated in the SC layer. The SC layer provided a motor command to the camera and sent a corollary discharge of this command to the FEF layer. Activity in the EP layer was updated to reflect the new position of the camera.
4. Changes in the BC layer cause an arm movement. The spatial error between where the arm points and where the ball is located, in the workspace reference frame, provided quantification of performance. The goal was to minimize this error at all times, regardless of whether the ball or the camera moved.

From the robot's “point of view,” the image of the ball would appear to move either if the ball moved, *or* if it only appeared to move due to saccadic rotation of the camera eye. Correct performance—continuous pointing to the ball—required the model to distinguish between real, external movements of the ball and illusory movement due to self-motion of the camera. If, due to external stimuli, the ball were to move in front of the robot, the hand should follow the movement of the ball. However, if a saccade were generated, the image of the ball on the retina would shift but the hand should remain stationary. If the robot were able to make this distinction between sensory changes caused by external (ball) movement vs. self-generated (camera) movement, it would imply that the network achieved useful visual stability despite eye movements.

### Training

At the start of a training run, the connectivity of the network was random where the weights could be small and positive or negative (–0.1 to 0.1). A supervised learning technique, backpropagation through time, was used to train the network (Rumelhart et al., 1988; Williams and Zipser, 1995). During training, a small amount of noise was injected into the updating of weights (~10^−5^) to prevent stagnation in local minima. Training took place in two stages. First, the connection from FEF and EP to BC was trained to perform a spatial vector addition thus creating a body-centered coordinate. These weights remained unchanged thereafter. Second, the core model (Figure 1A) was trained by back-propagating the error through the network and only modifying weights in the core. Specifically, the only error driving the updating was defined as the difference (in pointing workspace coordinates) between where the output of the model predicted the object to be as compared to where it truly was. When the changes in weights were minimal, approximating an asymptotic steady state in the network, the training was stopped.

During the training of the feedforward network, the target could appear in one of 25 locations evenly spaced within the central region of the Retina layer (5 × 5 grid, –20° to 20° by steps of 10°). An analogous input set of 25 evenly spaced locations was used on the SC layer corresponding to saccade vectors of ±20°. The recurrent network was trained on a smaller set of inputs comprising five retinal locations (horizontally: –20° and 20°; vertically: –20° and 20°; and one at the center) and four saccade vectors (horizontal saccades: –20° and 20°; vertical saccades: –20° and 20°). This yielded 20 unique combinations of trials. To maintain good coherence between the feedforward and recurrent networks, only the same 20 input combinations were analyzed in the feedforward network.

A single trial consisted of 17 time points of 10 ms each. For the first 50 ms, the target was presented at the presaccadic location and there was no CD input. For the next 70 ms, the SC layer signaled the upcoming saccade while the target remained immobile on the Retina layer. Finally, for the last 50 ms, the activity in the SC layer was quenched and the target was presented at the postsaccadic location.

## Results

### Training the core of the model with and without CD

To explain the changes in the model during training as clearly as possible, we will start by describing what happens if only the central elements of the core model are used (Figure 1A). Using only feedforward connectivity between neural sheets, the relative transmission delays between them need to be specified. We hard-coded the following biologically based latencies (using 10 ms increments) into the network: visual afference lag (Retina to Hidden layer) of 70 ms (Nowak et al., 1995), CD pathway delay (SC to Hidden) of 10 ms (Sommer and Wurtz, 2004a), and efferent delay (SC signal to saccade initiation) of 50 ms (Ottes et al., 1986). We compared the outcome of training the system with and without CD input to the Hidden layer. To eliminate CD input, we set its weights at the Hidden layer to zero.

**Figure 2:**
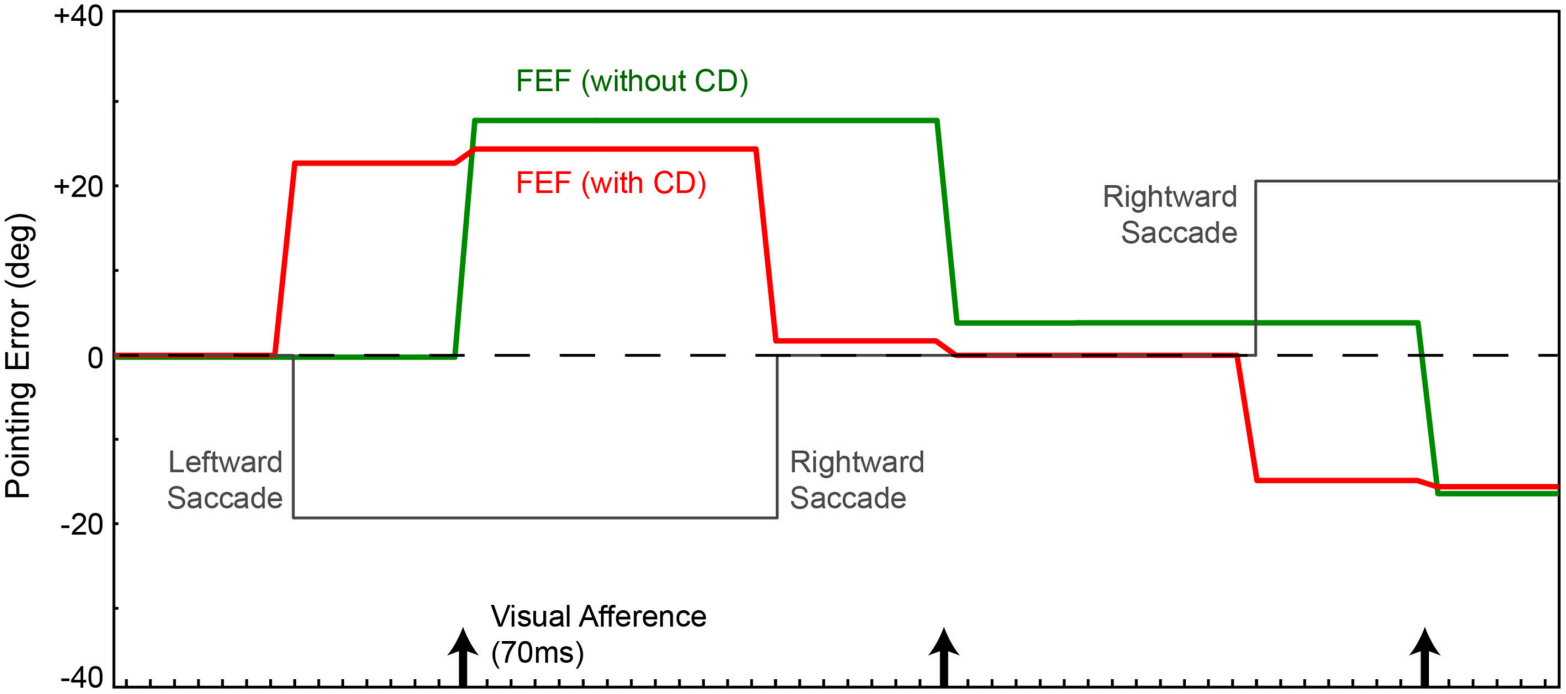
**Pointing errors as a result of saccades using the network in Figure 1A**. Ordinate is error, the angle between arm direction and the visual stimulus, i.e., the red ball, in workspace coordinates. The ball location is at zero in this representation (horizontal dashed line); ideal performance would match this line. Positive error is a rightward deflection of arm relative to ball. Abscissa is time. In this experiment, for illustration of the output signal in FEF, the FEF layer directly controls the arm (Figure 1A network, no BC layer). In a visual-only configuration, with no corollary discharge (CD), activity in the FEF and therefore arm position (green trace) change only after the visual afference lag, i.e., when new visual information arrives after the saccade. The arm moves opposite to each saccade (gray trace). With CD added, activity in the FEF and thus arm position (red trace) change predictively, around the time of the saccade, well in advance of the visual afferent lag. Because the FEF output is in retinal coordinates, with no accounting for camera position relative to the arm, the robot always has large errors in performance.

We focus on the activity of simulated neurons in the FEF sheet. Those neurons are visually-responsive due to input from the retina-dorsal visual stream input, and they are topographically arranged to match their receptive field locations. For example, if the stimulus (red ball) is located to the right on the retina, FEF neurons to the right of center will be active (analogous to “firing” in real neurons). Using the network without CD, every time the robot made a saccade (Figure 2, gray), the locus of neurons responding to the stimulus updated only after the visual latency of 70 ms, when new visual information arrived at the FEF (Figure 2, green). Note that a saccade in one direction causes the image on the retina to move in the equal and opposite direction, and thus the FEF locus of activity to move likewise. In this configuration, without CD, every saccade elicited a movement of the arm, which was inappropriate because the ball did not move in the arm's workspace. But in this simple model, pointing is based on retinotopic visual information alone, and so it is not possible to distinguish between actual ball movements and apparent movements due to saccades. The retinal changes are identical, and the FEF sheet has no information about the saccades. Given the feedforward nature of the network, changes in error occur instantaneously and are computed at each iteration of time.

If we use the same network, but provide information about saccades from SC to FEF (CD signals), the trained behavior improves temporally but not spatially. The robot's arm moves not after a visual afferent lag, but rather, as soon as the CD arrives. This causes the neural locus of activity in the FEF to remap predictively (Figure 2, red). The pointing is now better synchronized to the perturbing event (the saccade), which is good, but it remains locked to retinotopic spatial coordinates. For accurate pointing, the arm needs to know the stimulus location in body-centered coordinates, not retinal coordinates. Hence, the next step was to convert FEF output to a body-centered reference frame.

### Body-centered coordinates

In a real animal, retinal coordinates need to be transformed through a series of steps to reach a reference frame natural for the visually-guided effector, e.g., retinal to head-centered, then to body-centered. In our robots, this is simplified because the camera is fixed in the head, making the head effectively one big eye. Hence the necessary coordinate transformation for guiding the arm requires only one step, retinal to body-centered coordinates. This transformation is accomplished by including information about eye (= head/camera) position on the body. Shown in Figure 1B, an additional sheet was added that represented this eye position signal (EP layer). The spatial coordinate of the object in the FEF layer, combined with the eye position information in the EP layer (angle of eye/head/camera on body), produced a representation of the object in a Body-centered coordinate frame (BC layer) and thus an appropriate target for pointing. Based on studies of eye proprioception timing, we set the latency for the updating of eye position after a saccade to 50 ms (Xu et al., 2011).

**Figure 3:**
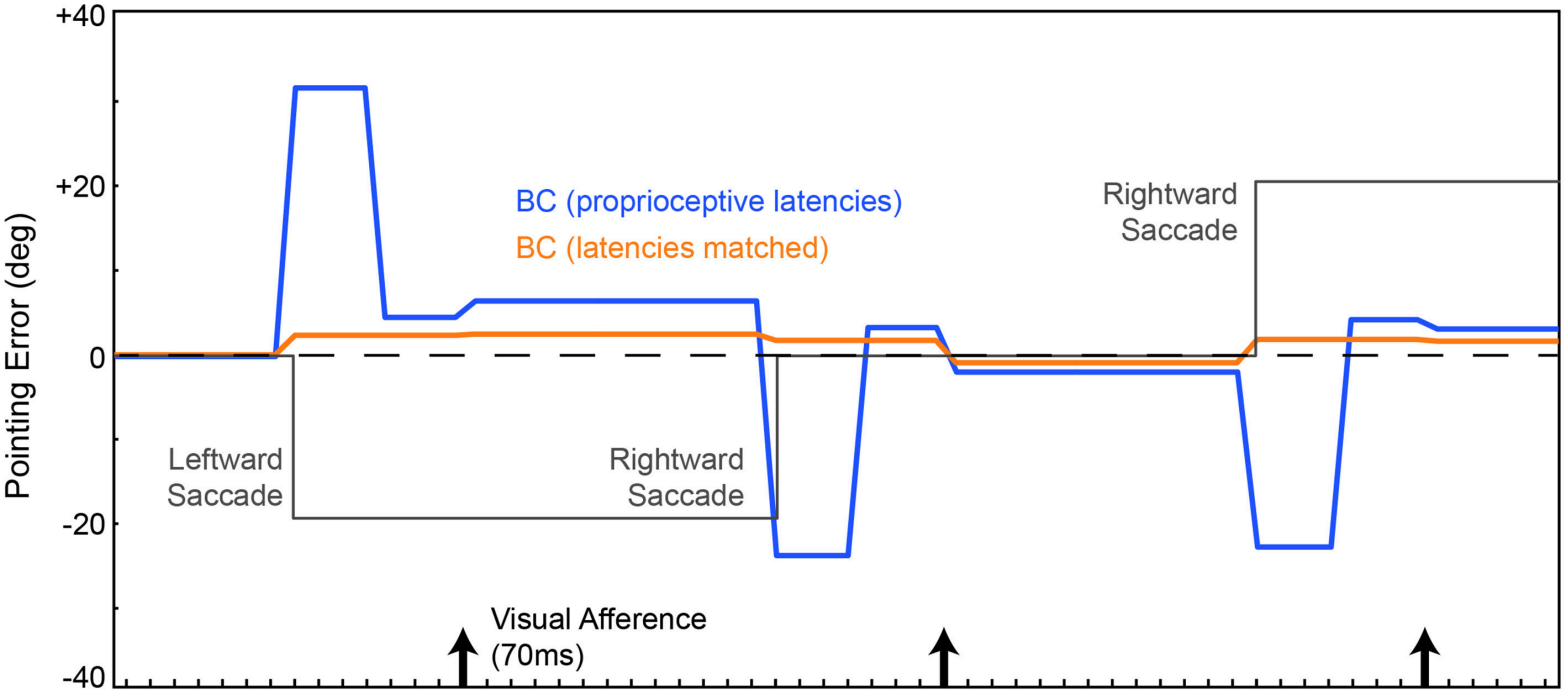
**Pointing errors as a result of saccades using the network in Figure 1B**. We are leaving out the recurrent connection in the Hidden layer at this point, so latencies are still hard-coded. FEF output is combined with eye position information in the EP layer to create a BC layer representation of the object that guides the arm. Conventions as in Figure 2. In one model (blue trace), the EP signal was updated at known proprioceptive latencies, 50 ms after the saccade. In a second model, the EP signal was updated in synchrony with the arrival of CD information at the FEF layer (orange trace). Both networks outperformed the models in Figure 2, due to the addition of the BC layer. And when the latency of the EP update matched the latency of the CD signal, pointing was quite accurate through the course of the trial, nearly matching ideal performance (dashed line).

The resulting network (Figure 1B) was trained from scratch, again using the metric of pointing error. CD was included. We are not including recurrent connections in the Hidden layer yet. On average through a trial, the network localized the ball more accurately (Figure 3, blue trace) than the previous instantiation (cf. Figure 2, green and red traces). Notably, however, the network briefly mislocalized the object around the time of the saccade, until postsaccadic visual afference arrived at the FEF layer. The small errors that remained after that are due to imperfections in the inverse kinematics used to drive the movement of the arm.

Around the time of the saccade, the network always mislocalized the object because the visual information in FEF was remapped by CD input prior to the proprioceptive updating of eye position. This is reminiscent of transient errors in perisaccadic target localization that are well-known from psychophysical experiments (Ross et al., 1997, 2001; Hamker et al., 2008). Though apparently similar, the mislocalizations seen in the model are likely unrelated to those psychophysically reported mislocalizations. The latter occur only under special circumstances. The stimulus has to be very briefly flashed stimulus, have a dimly lit background, and there has to be some permanent reference scale to which the stimulus is compared (Kaiser and Lappe, 2004). None of those conditions are relevant to our experiment.

Because proprioceptive eye position was inadequate for continuously accurate visual stability in our task, we tested what would happen if the EP signal changed predictively, essentially as a corollary discharge of eye position rather than eye movement vector. This is reasonable considering that the brain has internal signals about upcoming eye positions (Schlag-Rey and Schlag, 1984; Tanaka, 2007) and such signals influence cerebral cortex, albeit through pathways that are currently unknown. We altered the delays in the network such that the EP layer updated in synchrony with arrival of the CD signal at the FEF layer. Now, with matched latencies, the network performed admirably despite saccades disrupting visual input (Figure 3, orange). The network attained useful visual stability, operationally defined as continuously accurate pointing (aside from small, hardware-related errors).

### Emergence of shifting receptive fields

**Figure 4:**
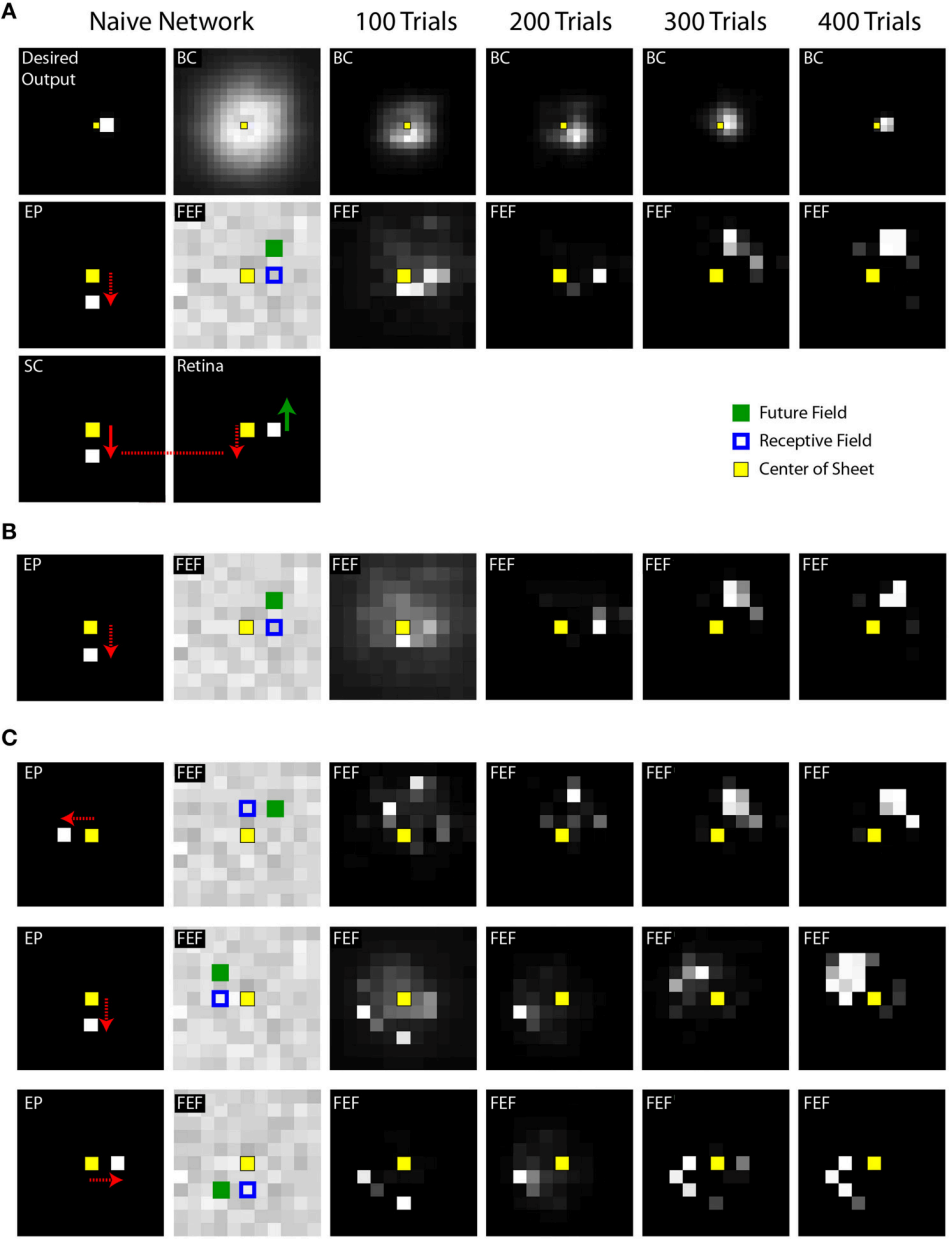
**Example training experiments. (A)** Each column shows a snapshot of activity during a single trial when the SC sheet (lower left corner) has produced its saccadic command (white square in the SC sheet with arrow showing vector of movement) but before the eye has begun to move. Specifically, the snapshots are taken 50 ms after the command and 20 ms before the movement. In this example trial, the eye is initially in the center of the sheet and the target is located, in retinal coordinates, to the right of the center. This is represented in the “Desired Output” sheet, in workspace coordinates, as a target just right of the center. A downward saccadic command would move the retina downward (red arrow) and thereby cause the image of the object to move upward on the Retina sheet (green arrow). The internal representation of eye position (EP) was updated simultaneously with the saccade command (as in Figure 3, “latencies matched” case). The weights from the Retina and SC to the FEF were initialized to be random in the naive network. Through training (moving rightward in the illustrations), the BC sheet gradually reached the Desired Output for this point in time in the trial, after saccade command but before eye movement (and all other points, not shown). During these training iterations, at this point in the trial, the FEF sheet goes through an early phase of activation focused on neurons that represent the approximate retinal location of the target (to the right of center), but then shifts to activation of neurons representing the location where the target will be (upward) after the saccade. In other words, training the system for spatial constancy caused the FEF to remap its visual representation of the target just before the saccade. **(B)** Training time course of a second network, using the same stimulus location and saccade vector, but different initial random weights in the maps. Shown is the same trial sequence as shown in panel **(A)**, but for brevity, only the middle row. **(C)** Three more examples using a variety of stimulus locations and saccade vectors. While the fidelity of the final pattern of sheet activity varies between outcomes (sometimes a punctate final representation, sometimes more dispersed), in all cases, the final centroid of neural activation showed presaccadic remapping, in that the Future Field location was better represented than the Receptive Field location at this point in the trial, just before the saccade.

A single trial of training or testing the model includes *behavioral* events, which we have focused on thus far, and concomitant *neural* events, which we describe now. The neural events include changes in activity patterns across the topographic maps of the Retina, SC, FEF, Hidden, EP, and BC layers. Figure 4A shows snapshots of the simulated neural activity in various layers of the model during learning. The “Desired Output layer” (Figure 4A, upper left corner) illustrates the activity in the BC layer that is needed to accomplish an accurate reach to the target. In other words, it represents where the target actually is in the workspace (in this case, to the right, requiring the arm to point to the right). For useful visual stability despite saccades, i.e., continuously accurate pointing, the activity in the BC layer needs to match that in the Desired Output layer throughout every trial despite changes in the SC layer (which moves the eye) and consequent changes in the Retina layer (which suddenly displaces visual input to the FEF).

The snapshots in Figure 4 are taken at a point in time during the trial when presaccadic remapping, if it occurs, should be prominent: after the SC layer commands a saccade but before the saccade begins. The activity patterns in this presaccadic period in the FEF and BC layers, after the 100th, 200th, 300th, and 400th training trials, are extended out to the right of Figure 4A. Before training (Figure 4A, Naive Network), the FEF and BC sheets are initialized with random weights and thus are noisy. After 200 trials of training, FEF neurons with receptive fields close to the object location (blue squares) are active during this presaccadic epoch. That is, there is no remapping at this point in training; the neuronal representation is essentially veridical and does not seem to take into account information about the upcoming saccade that is supplied by the CD signal. But after 300 trials of training, neurons that spatially correspond to the future field (green filled squares) begin to respond, and after 400 trials, this effect crystallizes. The network exhibits presaccadic remapping in FEF sheet space: just before the saccade, neurons that normally respond to stimuli at the upper right of the retina are representing a stimulus (the red ball) located straight right on the retina. And neurons that normally respond to the straight right location are inactive even though there is a stimulus in their receptive field.

**Figure 5:**
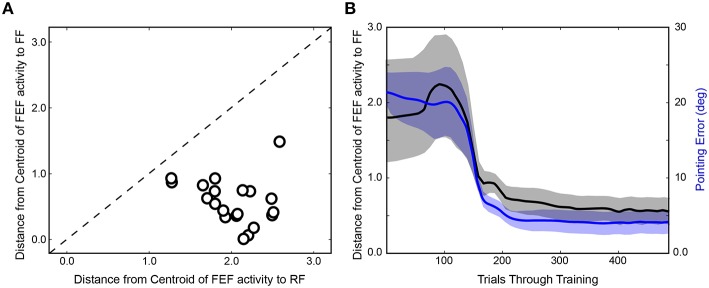
**Summary of training effects on FEF activity. (A)** Final prevalence of presaccadic remapping in the neurons. For the FEF sheet of the trained model (400th trial) during the presaccadic period, plotted is the distance from the centroid of neural activity to the Receptive Field (RF) and Future Field (FF) locations. All points lie below the unity line, indicating that neurons representing the FF were more active than those representing the RF. **(B)** Correspondence between emergence of presaccadic remapping and improvement in behavior. The left ordinate is the same as for panel **(A)** (distance from centroid of activity to the FF location in the FEF sheet), but now this centroid location (black) is plotted across training and compared with the errors in pointing (blue; right ordinate). Each trace shows mean ± SE over the 20 training runs. On average, after about 100 trials of training, remapping began to emerge (plummeting blue curve). In synchrony, pointing errors dropped drastically.

After completing the first run of training (Figure 4A), the network was reinitialized to random weights and retrained. In Figure 4B, the same trial sequence was plotted but showing a slightly different evolution of FEF activity patterns that nevertheless led to similar presaccadic remapping after 400 trials. The training time courses for three additional stimulus-saccade configurations are shown in Figure 4C. Each of these examples shows similar patterns of early, near-veridical representation of the stimulus followed by final, remapped representation of the stimulus, during these snapshots of the presaccadic period. The spatial cohesiveness of the final remapped representation varied between examples, sometimes yielding a distinct location and sometimes a more “scattered” pattern (compare FEF sheets across rows after 400 trials in Figure 4), but the centroid of this final representation was always much closer to the Future Field than to the Receptive Field (Figure 5A). The development of this presaccadic remapping effect corresponded tightly with improvement in performance (decrease in pointing errors) during training (Figure 5B).

**Figure 6:**
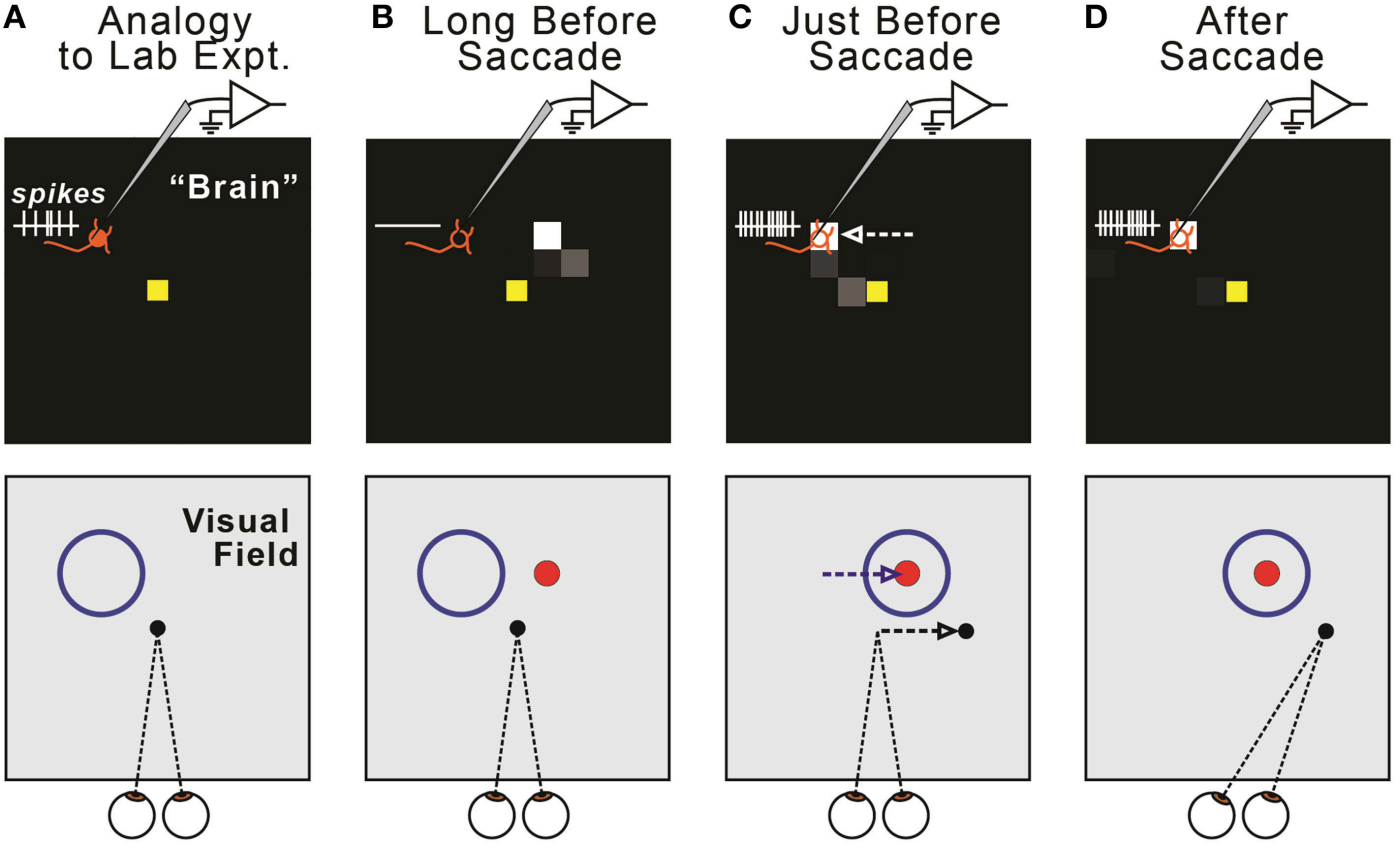
**Relationship between remapping of neural activity in the model (top row) and remapping of visual sensitivity in the visual field (bottom row). (A)** The model displays all FEF neurons, each of which has a Receptive Field (RF). We quantify changes in the locus of activity across the sheet (“neural remapping”). In contrast, neurophysiologists typically record a single neuron and quantify changes in its visual sensitivity across the visual field (“receptive field remapping”). The one-to-one correspondence between neural and receptive field remapping may not be obvious. It helps to imagine recording from a single neuron in the modeled “brain” (top) as a monkey makes fixations and saccades (bottom). Due to our sheet topography, a neuron located up-left in the sheet (top, orange “neuron” icon) has a receptive field up-left from the point of fixation (bottom, blue circle). **(B)** Long before a saccade, the recorded neuron exhibits no activity (top), because the visual stimulus (red ball, shown as a dot) is outside of its classical RF (bottom). **(C)** Just before a rightward saccade, the centroid of active neurons shifts leftward on the FEF map (top, white arrow). A physiologist would observe that the “recorded” neuron now responds to the red ball stimulus; that is, its visual sensitivity has shifted rightward (bottom, blue arrow), parallel to the upcoming saccade vector (bottom, black arrow). In general, in our model, presaccadic remapping of neurons on the FEF sheet implies oppositely directed presaccadic remapping of visual sensitivity in the visual field. **(D)** After the eye moves, the subject fixates a new location, and the neuron's visual sensitivity is now back at its classical receptive field.

This presaccadic remapping is a replication of the physiological result that has been reported numerous times (e.g., Duhamel et al., 1992; Walker et al., 1995; Nakamura and Colby, 2002; Sommer and Wurtz, 2006). In Figure 4, the remapping was shown as a function of neural location in the FEF sheet, but this corresponds directly to the remapping effect that is more familiar to neurophysiologists, a change in location of visual sensitivity—from receptive field to future field—for a single, recorded neuron (Figure 6). Presaccadic remapping in both places, the neural space of the modeled FEF sheet and the visual field of a subject's workspace, depends on the vector of the upcoming saccade, but with opposite directional relationships (Figure 6C): the remapping is *antiparallel* to the saccade vector in the FEF sheet but *parallel* to the saccade vector in the visual field.

**Figure 7:**
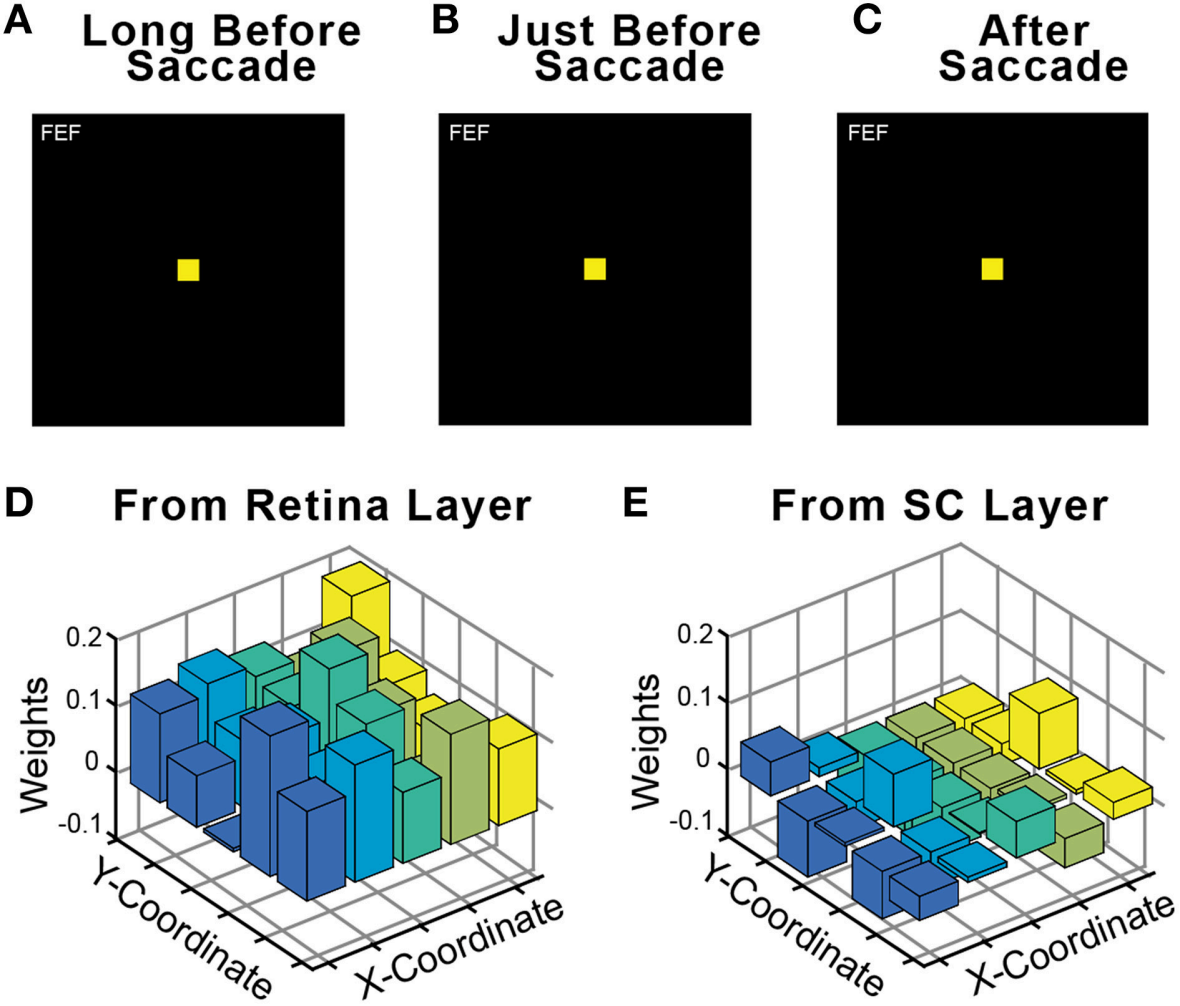
**Relative influence of visual vs. CD signals in the FEF sheet**. During training, a visual stimulus was always present on every trial. Fully trained networks were then tested in a no-stimulus condition, which were normal trials except that the visual stimulus was omitted. As shown for this example trial, in the absence of a visual stimulus the FEF sheet showed no activity **(A)** long before the saccade, **(B)** just before the saccade even though CD activity was present, and **(C)** after the saccade. Consistent with FEF activity driven by visual driving input but modulated by CD input, the weights of connections onto the Hidden layer were much higher from **(D)** the Retina sheet (average 0.113) than from **(E)** the SC sheet (average 0.046).

An important control in neurophysiological studies of presaccadic remapping in the FEF is to repeat the task, but with no visual stimulus present. This reveals the extent to which non-visual factors, such as motor planning to generate the saccade or the impact of CD signals, contributes to the activity of the neuron. Ideal presaccadic remapping is a visual response and should disappear in the absence of the visual stimulus. We performed this control in our trained model and found that with the visual stimulus removed, there was no activity in the FEF sheet (Figures 7A–C). Thus, all neural activity in the FEF sheet were visual responses. An interesting implication of this result is that the CD signals in the model did not directly drive activity in the FEF layer neurons, even though they clearly caused the presaccadic shift in the visual response of those neurons (for supporting evidence of this, see CD inactivation studies, below). The CD signals act as a modulator of activity. We examined the weights from the central 25 neurons that were trained (see Section Training; exploring the full development and precise configurations of weights in the Hidden layer and the recurrent connections that yield this outcome is beyond the scope of this report). Consistent with a modulator hypothesis of CD input, we found that the weights of the CD afferents were less than half the magnitude (41%) of the weights of the visual afferents in the trained model, and often were negative, while all visual weights were positive (Figures 7D,E). This conclusion matches the findings of *in vivo* inactivations of the SC-MD-FEF pathway in monkeys, which cause deficits in operations that require corollary discharge (inaccurate second saccades in a double-step task, (Sommer and Wurtz, 2002); reduction of future field activity in FEF neurons, (Sommer and Wurtz, 2006); impaired stability of visual perception, Cavanaugh et al., 2016) while sparing the generation of individual saccades and nearly all other measured parameters (unaffected receptive field responses in FEF, (Sommer and Wurtz, 2006); intact visual, working memory, and saccade performance except for a slight omnidirectional increase in reaction time, (Sommer and Wurtz, 2004b).

### Learned timing using recurrence

Thus, far, we have used a fully feedforward model. Next we remodeled the network architecture to discover optimal relative timings instead of explicitly providing them in connections. We removed the delays between the Retina layer and the SC layer to the Hidden layer and added a recurrent connection within the Hidden layer (see Figure 1B). The one latency that we controlled explicitly was that of the signal that is combined with the final output of the FEF sheet, the timing of updated eye position information (in the EP layer). Again, the only training criterion was that the Body-centered representation of the object remain stable through the course of a trial, thus minimizing pointing errors.

**Figure 8:**
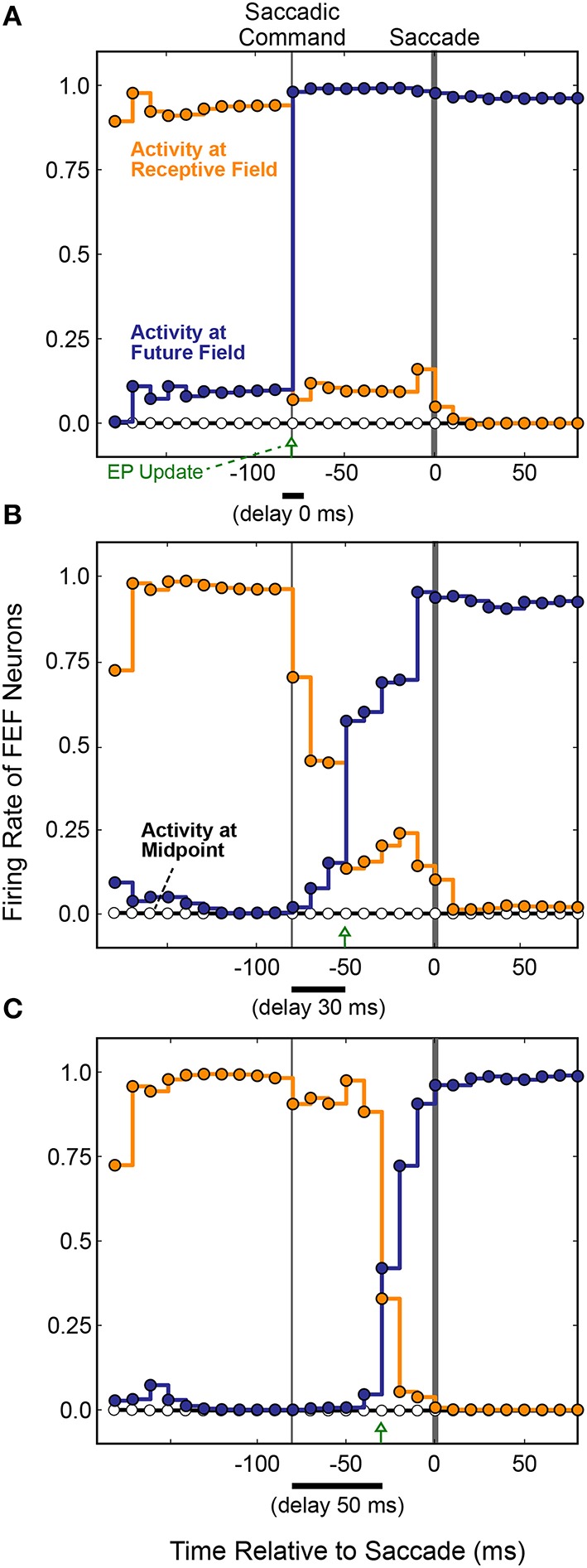
**FEF remapping dynamics in the full, recurrent model**. Initiation of the motor command in the SC layer is labeled “Saccadic Command.” After an efferent delay of 70 ms, exaggerated to provide detailed analysis of the presaccadic period, the saccade occurred (time 0 on the abscissas). Each dot shows the state of activity in the FEF layer as it was updated in 10 ms intervals for neurons representing the Receptive Field (RF; orange), the Future Field (FF; blue), and a midpoint location between the RF and FF (white). Lines connecting the dots indicate the steady activity level at each FEF location until each update. The timing of the EP update is delayed relative to the Saccadic Command by **(A)** 0 ms, **(B)** 30 ms, and **(C)** 50 ms. Remapping was time-locked to the EP updates and jumped rather than spread, as there was no transient activity at the midpoint.

The first main result was that presaccadic remapping still occurred: just before the saccade, the locus of activity in the FEF layer shifted from neurons representing the receptive to neurons representing the future field. To observe the details of dynamics during the presaccadic period, we expanded it by delaying the saccade to occur 70 ms after the initiation of the motor command in the SC layer. Figure 8 summarizes the results when the model was trained with three different EP delays (0, 30, and 50 ms after the SC saccade command, marked with green arrows). Regardless of the EP delay, activity at the Receptive Field went down (Figure 8, orange) and activity at the Future Field went up (Figure 8, blue) before the saccade.

The second main result was that the remapping tracked the EP latency. As the EP update was delayed (Figures 8A–C), remapping in the FEF was delayed proportionately in the trained models. This makes sense, conceptually, because for the BC output (and thus pointing accuracy) to remain stable, the FEF output must counter the EP signal precisely when it changes. Otherwise, the system combines current information about where the stimulus is on the retina with predictive information about where the retina will be oriented. To prevent mislocalization of the stimulus, the FEF representation of where the stimulus is on the retina must be shifted predictively, opposite to the predictive EP signal.

Also plotted in Figure 8 is the activity of a neuron midway between the Receptive Field and the Future Field (black horizontal line and white circles). Activity at this midpoint location would suggest a spread of remapping, as opposed to a “jump,” from the Receptive Field to Future Field neurons. In the lab (Figure 6), this would correspond to a spread of visual sensitivity between the Receptive Field and the Future Field in visual space, as found in one study for a parietal cortex region (Wang et al., 2016). As indicated by the flat Midpoint activity lines in Figure 8, there was no such spread in our model. Remapping in the simulated FEF sheet involved a jump in activity from the Receptive Field to the Future Field as found for the real FEF (Sommer and Wurtz, 2006) and reported in numerous other physiological and computational studies (Duhamel et al., 1992; Walker et al., 1995; Umeno and Goldberg, 1997; Nakamura and Colby, 2002; Keith and Crawford, 2008; Keith et al., 2010). The jump in activity from FEF neurons representing the Receptive Field to those representing the Future Field was not instantaneous, however. Rather, there was a gradual ramping down of activity at the Receptive Field and up at the Future Field. Similar dynamics were observed physiologically (Kusunoki and Goldberg, 2003).

### Lesioning CD input

**Figure 9:**
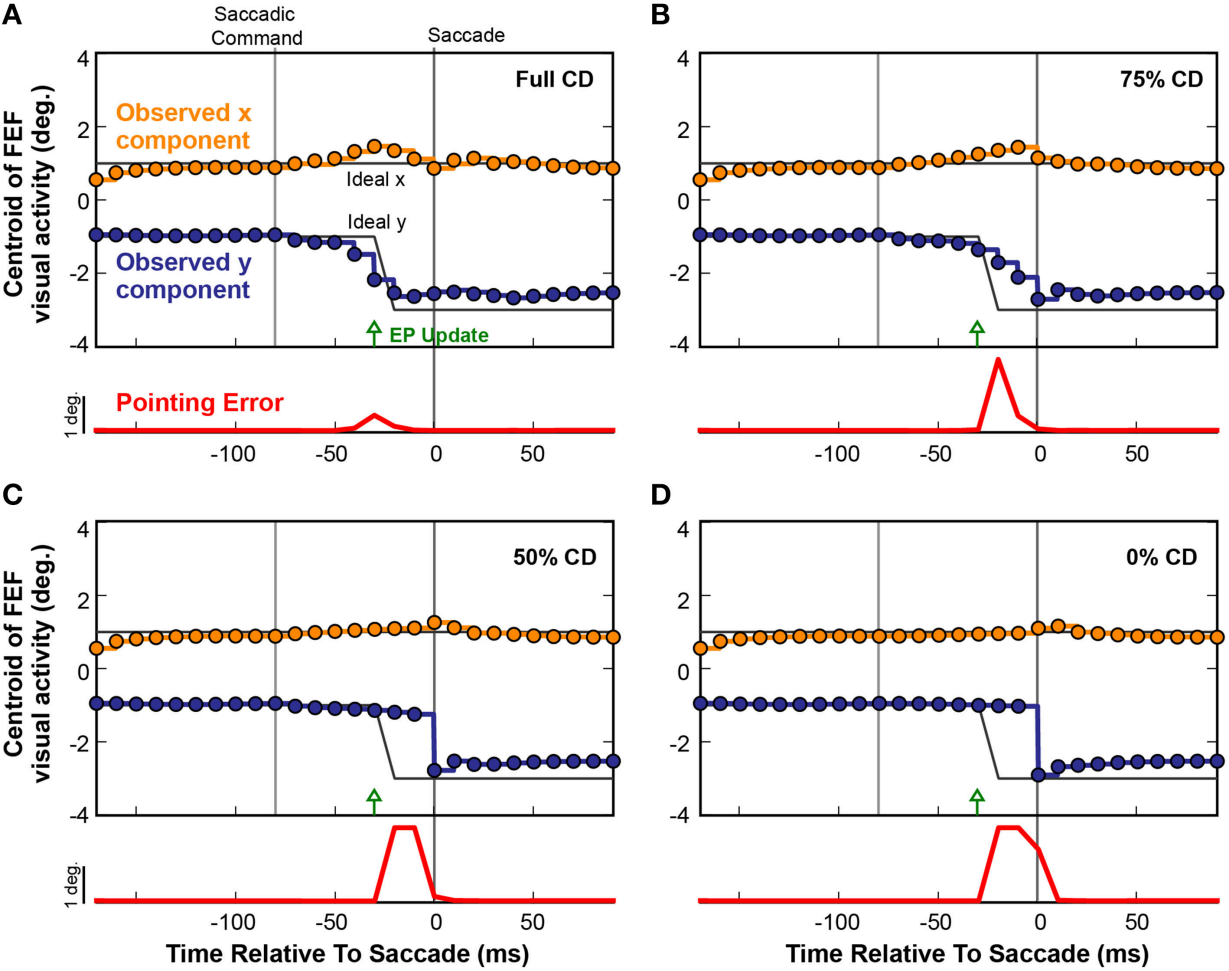
**Four repetitions of a single trial as CD was reduced from (A) fully intact CD, to (B) 75%, (C) 50%, and (D) 0% of the intact level**. Each circle represents the centroid of FEF activity (x component, orange; y component, blue) at each refresh of the FEF layer (every 10 ms). Activity between the updates is shown by same-colored lines. The black lines denote the ideal movement of the centroid with this saccade-stimulus configuration, such that the error in BC coordinates would stay at zero and arm pointing would be continuously accurate despite the saccade. The red trace shows errors in pointing at each time point. As the CD pathway was silenced in successive trials from 100% (normal) to 0% (full shutoff), i.e., from panels **(A–D)**, the remapping became more gradual and started later. Even with just 50% reduction in CD strength **(C)**, the presaccadic remapping disappeared and the error in the network, and thus in pointing (localization of the stimulus), became large.

To causally test the contributions of CD on visuomotor behavior, presaccadic remapping, and visual perception in monkeys, laboratory studies used injections of muscimol (a GABA_A_ agonist) into the thalamus to temporarily inactivate the SC-to-FEF pathway (Sommer and Wurtz, 2004b, 2006; Cavanaugh et al., 2016; see also Tanaka, 2006). Analogous “inactivations” can be applied to neural networks to systematically test the influence of specific connections (White and Snyder, 2004, 2007). To inactivate the CD pathway in our model, we systematically scaled down the weights between the SC layer and the Hidden layer to decrease CD information. Figure 9 shows the effect of such an inactivation experiment on a single saccade in the fully trained recurrent network. Instead of plotting activity at the discrete Receptive Field and Future Field locations as in Figure 8, here we follow the FEF activity continuously by plotting its centroid (separated into x and y components relative to the FEF map). The horizontal gray lines in Figure 9 depict the ideal remapping that would keep pointing errors at zero. With an intact CD connection (Figure 9A), activity in the FEF started to remap just prior to the EP update and finished just prior to the saccade. Though not shown explicitly here, this occurred as in Figure 8 by a drop in activity at the Receptive Field and an increase at the Future Field with no change in activity in between (although the centroid passed in between because it is the spatial average).

When CD was reduced to 75% of the original strength (25% loss in magnitude of the SC-Hidden layer weights; Figure 9B), the remapping process began slightly later and did not complete until the saccade occurred and retinal information was updated. As we reduced CD further, the remapping was almost abolished. At 50% (Figure 9C), there was only a very slight movement of the centroid but no obvious presaccadic remapping. When the CD pathway was fully severed (Figure 9D), activity in the FEF layer remained unchanged until the saccade was executed, at which point, afferent visual information updated the FEF to signal the postsaccadic location of the visual stimulus. In each of the four experiments in Figure 9, the red traces show the Pointing Error. As CD strength decreased, Pointing Error increased in the period before the saccade, because the FEF continued to represent the presaccadic location of the object after the EP layer updated. The Pointing Error was transient because, as soon as the saccade was completed, it was resolved when afferent visual information combined with the updated EP signal.

**Figure 10:**
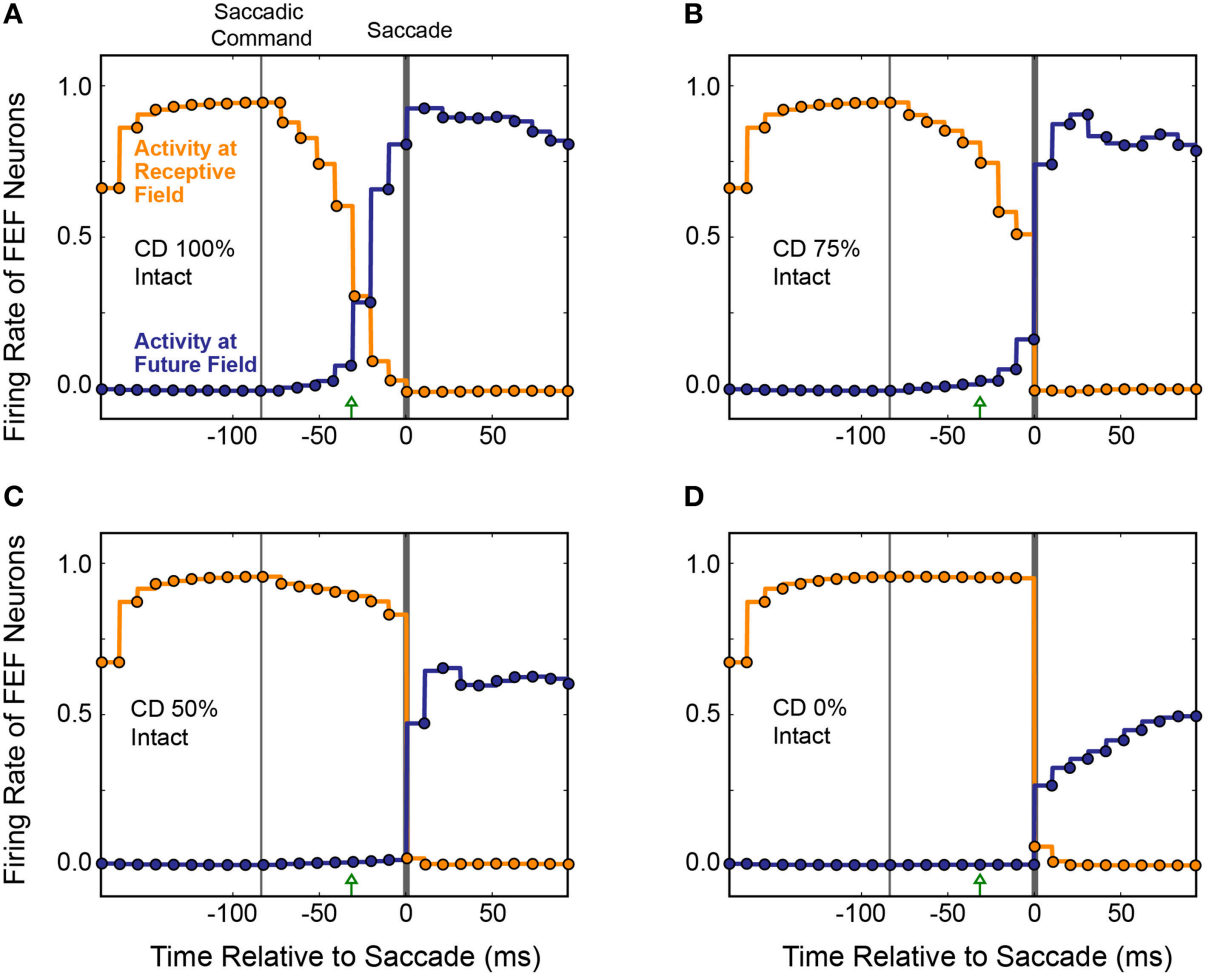
**Overall results of inactivating CD in the model**. Conventions as in Figure 8C. **(A)** With intact CD, presaccadic remapping began when activity was generated in the SC layer to produce a CD signal. Remapping was underway at the time of the EP update and was completed by the time the saccade occurred. **(B–D)** As CD was inactivated systematically, the time course of remapping became delayed. Without any CD **(D)**, neurons with their Receptive Field on the stimulus remained active through the perisaccadic window. The low activity of “Future Field” neurons after the saccade was an artifact of history within the network.

The average results from 20 simulated CD inactivation trials are shown in Figure 10. These experiments involved a variety of stimulus locations and saccade directions, so there was no common, ideal shift in the centroid of activity that would indicate remapping. Thus, in Figure 10 we return to the conventions of Figure 8 to show the activity at the Receptive Field and the Future Field of each experiment. With fully intact CD (Figure 10A), at the start of a trial, the average activity of neurons with their Receptive Field on the stimulus ramped up quickly while activity of neurons with their Future Field on the stimulus remained silent. Once the SC layer started signaling the impending saccade with CD, activity (visual responsiveness) of the “Receptive Field” neurons ramped down and, on a similar time scale, activity of the “Future Field” neurons ramped up. This presaccadic remapping was complete at the time the saccade occurred and, postsaccadically, activity of the “Future Field” neurons (now with their receptive fields on the stimulus) was sustained. At 75% of the original CD strength (inactivation by 25%; Figure 10B), the onset of the remapping (i.e., activity of the “Future Field” neurons) was delayed, as was the decrease in activity of the “Receptive Field” neurons. At 50% CD (Figure 10C), presaccadic remapping was virtually abolished.

It may appear in Figure 10 that as the remapping process was hindered, the postsaccadic visual activity was affected as well. This was an artifact of our recurrent network architecture, however. Postsaccadic visual activity was determined not only by updated visual input, but also by the previous time points. Because of this characteristic of the model, as CD inputs weakened, the postsaccadic visual activity (of “Future Field” neurons) could not ramp higher than half of its original activity.

**Figure 11:**
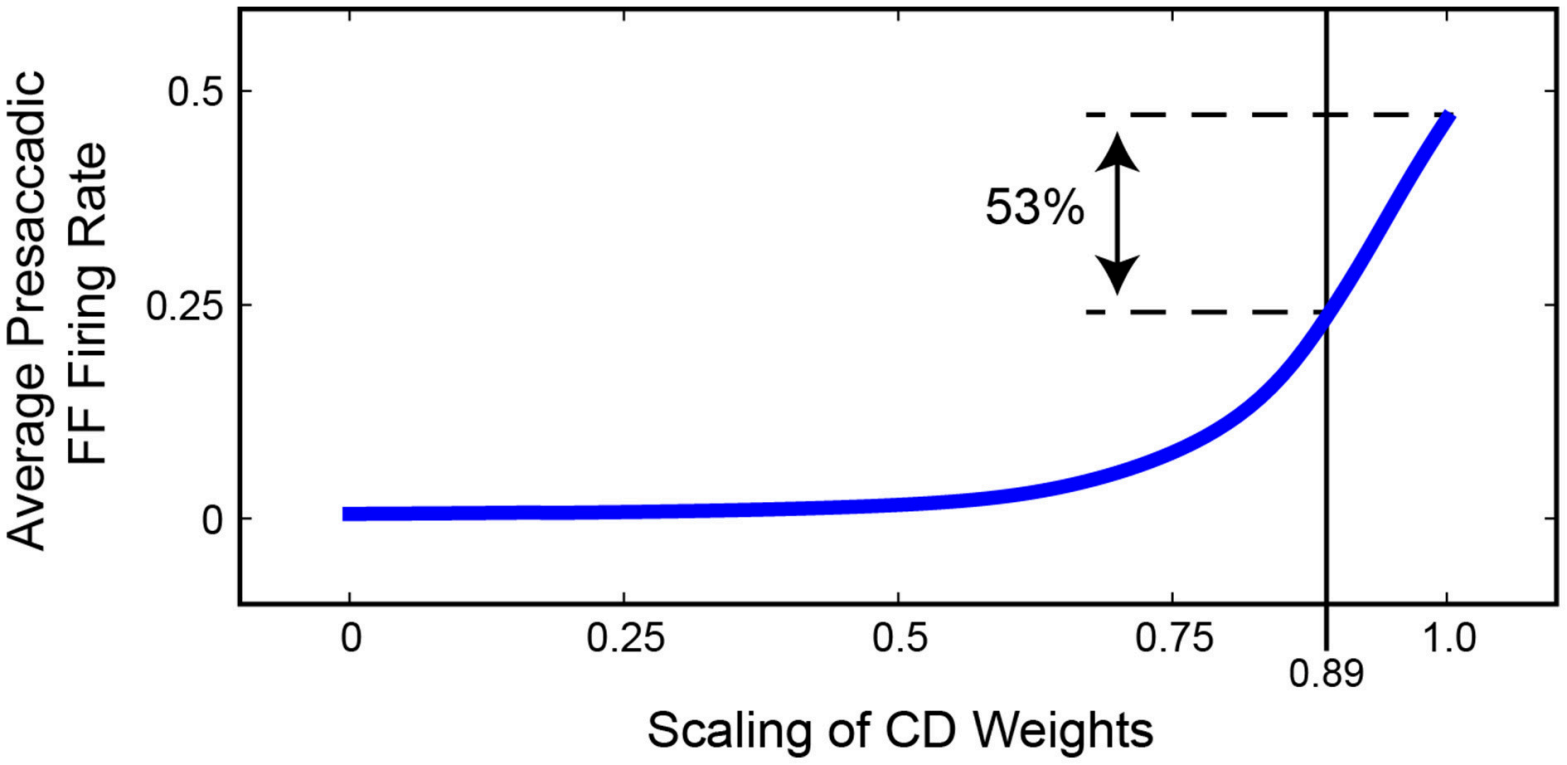
**Summary of the relationship between strength of FEF remapping and strength of CD in our model**. Plotted is the average activity of “Future Field” (FF) neurons during the 50 ms epoch leading up to the saccade. As the weights between the SC layer and the hidden layer are decreased (to left), the average presaccadic FF activity also decreases, although non-linearly. A 53% reduction in presaccadic remapping as found by Sommer and Wurtz (2006) would correspond to a 0.89 scaling of CD weights, that is, an 11% reduction of CD strength.

Figure 11 summarizes the relationship between presaccadic remapping and strength of CD in the trained, recurrent model. The curve quantifies the average firing rate of neurons with their Future Field on the stimulus over the 50 ms epoch leading up to the saccade. As the weights from the SC layer, i.e., CD input, decreased (toward the left on the x-axis), the amount of remapping decreased. Notably, however, the relationship was markedly non-linear: even small losses of CD caused large drops in the activity of “Future Field” neurons during the presaccadic epoch. The exact shape of the curve likely depends on the coefficients of the sigmoidal transfer functions in the model, but the point is that strength of remapping does not necessarily provide a linear readout of strength of the underlying CD signal. This helps in interpretation of *in vivo* results. Sommer and Wurtz (2006) found that inactivation of thalamus led to a 53% deficit in presaccadic remapping in FEF neurons, but from the model we see that this does not necessarily reflect the amount of CD lost. A 53% reduction of remapping in our model (i.e., activity of “Future Field” neurons during the presaccadic epoch) corresponds to only an 11% reduction in CD. A literal reading of this modeling result is that the pathway studied by Sommer and Wurtz (2006) conveys roughly 11% of the CD used by the FEF. Interestingly, this is the amount inferred from an earlier result of inactivating the same pathway using the double-step saccade task as the indicator of CD loss (Sommer and Wurtz, 2002), suggesting that the double-step task may provide a more sensitive, linear assessment of CD integrity than provided by the amount of remapping in FEF. To test the extent to which this result depended on model-specific parameters, we varied the sigmoidal transfer functions used in the network during propagation and found qualitatively similar results. Lesioning the CD input always caused a non-linear decrease in remapping. The amount of CD loss that yielded a 53% reduction in presaccadic remapping varied between 10 and 35%.

### Lesioning retinal input

**Figure 12:**
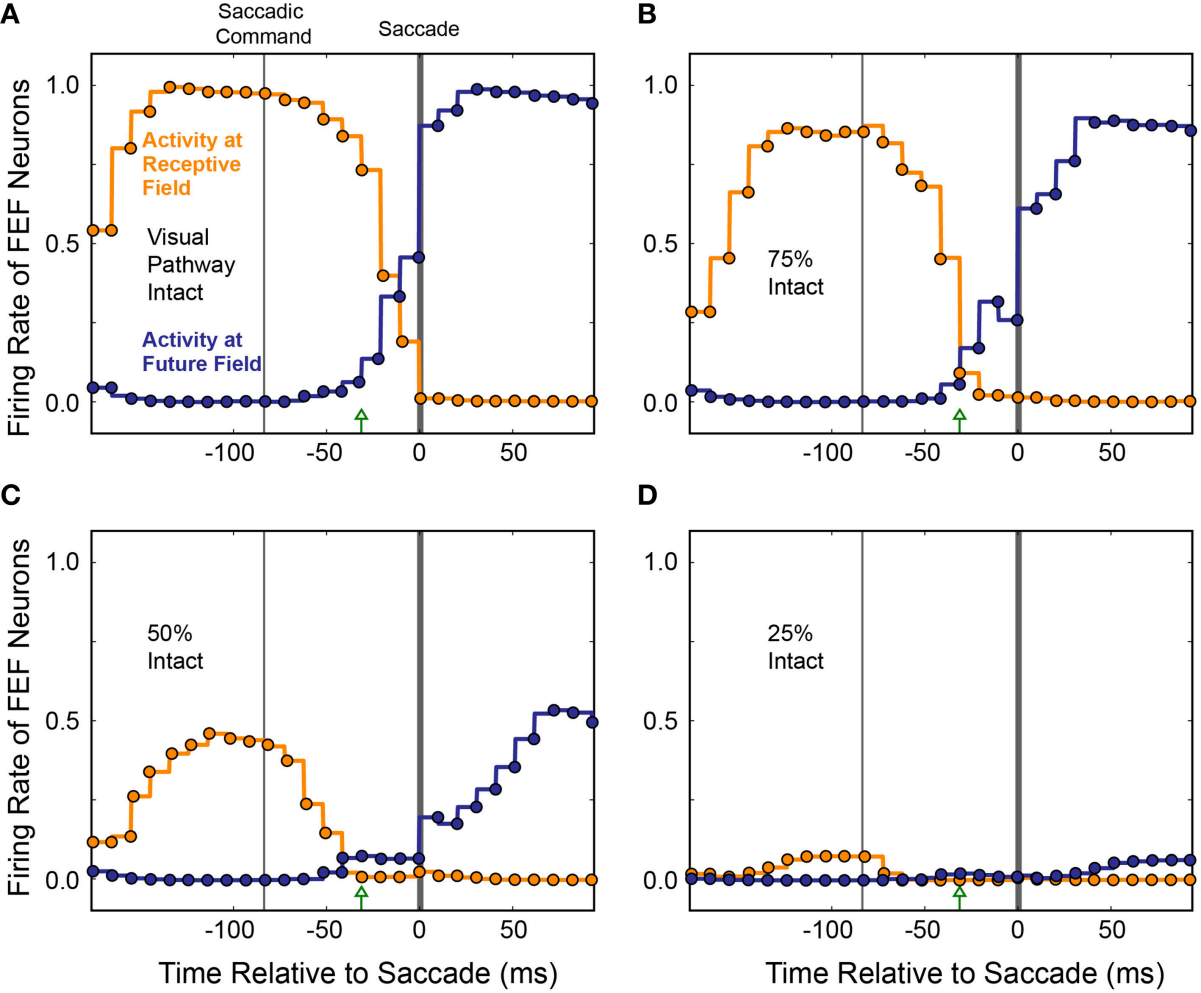
**Average activity of neurons with their Receptive Field or Future Field at the stimulus location across 10 experiments as visual input was decreased**. To simulate inactivation of visual input, weights from the Retina to the Hidden layer were systematically scaled down **(A–D)**. Visual responses decreased for both the “Receptive Field” and “Future Field” neurons, but presaccadic remapping was maintained, unlike in the CD inactivation experiments (cf. Figure 11).

To see if the loss of presaccadic remapping found for CD “inactivation” in our model was specific to loss of CD, or was a more general consequence of reduced input to the Hidden layer, we performed the same inactivations on visual input while holding CD strength constant at 100%. As visual input was reduced systematically, activity of both the “Receptive Field” and “Future Field” neurons decreased in tandem, with no change in their “crossover” point before the saccade (Figure 12). At 75% visual input, responses of both “Receptive Field” and “Future Field” neurons were more sluggish and slightly weaker at their peaks. At 50% visual input, the activity of both “Receptive Field” and “Future Field” neurons was reduced by about half and, at 25% visual input, it was nearly abolished. Nevertheless, at 75 and 50% visual input, presaccadic remapping at the Future Field persisted; the activity of the “Future Field” neurons began soon after the Saccade Command in the SC and well before saccade initiation. At 25% visual input, there was so little activity that this timing could not be assessed. While presaccadic remapping has not yet been studied *in vivo* during partial inactivations of visual input, these modeling results make testable predictions about what such an experiment would reveal—notably, no change in the occurrence or timing of remapping, even as neuronal visual responses in the receptive field and future field drop considerably.

## Discussion

In this report, we modeled the problem of maintaining a stable visual representation of objects across saccades. Our approach was to use a sheet-based, hierarchical neural network architecture with a recurrent hidden layer and biologically-based connections. As we trained the model to achieve useful visual stability, presaccadic remapping of visual receptive fields emerged. The remapping was synchronized to the updating of eye position and depended non-linearly on a corollary discharge of eye movements.

### Maintaining visual stability

The problem of maintaining visual stability across saccades derives from the slow afferent lags of the visual system. When the eyes move, renewed visual information takes about 70 ms to arrive in extrastriate cortex. The physical state of the eyes is out of register with the internal state of the visual system during this delay. To resolve this mismatch, information about the new state of the eyes could be delayed to match arrival of the visual input. Alternatively, the internal state of the visual system could be updated predictively to match the new state of the eyes. The primate brain achieves the latter, faster solution (Duhamel et al., 1992; Sommer and Wurtz, 2008).

Previous studies have focused on corollary discharge of saccades as the key to understanding predictive visual updating. A pathway for corollary discharge was identified (Lynch et al., 1994; Sommer and Wurtz, 2002), shown to convey predictive information about impending saccades (Sommer and Wurtz, 2004a), and confirmed as contributing to presaccadic remapping in the FEF (Sommer and Wurtz, 2006). Our present modeling results suggest that an additional predictive signal, representing eye position, is critical. This does not refute the contribution of corollary discharge of saccades. Visual signals must combine with corollary discharge of saccades to achieve remapping, and the inactivation studies in our model support this. Corollary discharge of saccades explains *how* remapping is created. In contrast, predictive eye position signals explain *why* remapping is created, and *when* it occurs.

**Figure 13:**
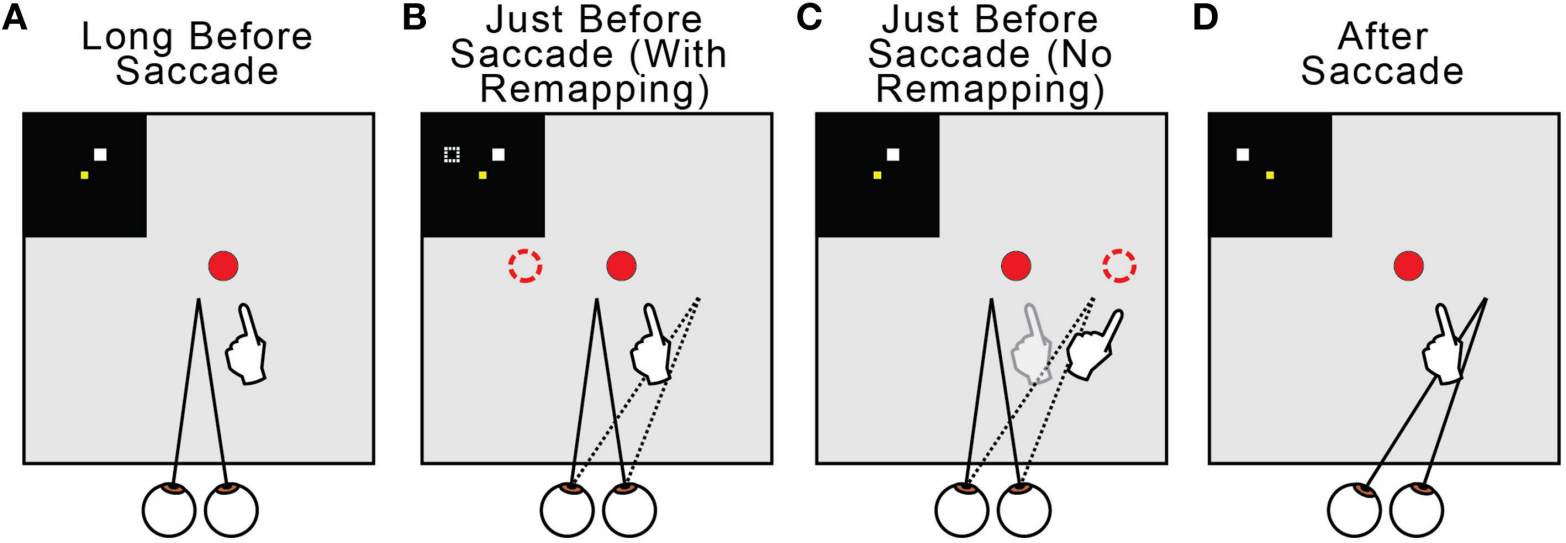
**Summary of presaccadic remapping for useful stability**. Solid lines denote actual positions and dotted lines the internal representations of positions. Insets depict activity in the FEF sheet. Hands show pointing behavior in the workspace, i.e., output of the BC sheet. **(A)** Visuomotor stability, i.e., accurate pointing, long before a saccade. **(B)** With presaccadic remapping, activity in the FEF sheet (dashed square in inset) represents the target at a new location (dashed circle), but when summed with predictive eye position signal (dotted line), the target representation in the workspace is stable and pointing is accurate. **(C)** With no presaccadic remapping, activity in the FEF sheet is unchanged. It is referenced to the updated eye position, causing an apparent shift in the target position (dashed circle) and inaccurate pointing. **(D)** Visuomotor stability after the saccade. Visual input from the new eye position has arrived.

Regarding the why of remapping, our model indicates that it acts to counter predictive eye position signals. Figure 13 summarizes this overall interpretation by illustrating the neural and behavioral events that accompany a rightward saccade. Long before the saccade (Figure 13A) and long after (Figure 13D), visuomotor stability, measured as accurate pointing to a visual target, is accurate. Just before the saccade, the internal estimate of eye position updates predictively to its rightward, postsaccadic location (Figures 13B,C, dotted lines). If remapping occurs (Figure 13B), the visual representation of the target updates leftward in FEF neural space (dashed square in the inset and dashed circle in the visual field). This leftward retinotopic signal is referenced to the rightward eye position signal and the representation of the target in the workspace is maintained. Neurophysiologists should keep in mind that, during a neural recording experiment, this shift in neural space would correspond to an opposite (rightward) shift of visual sensitivity in visual space (to the future field; recall Figure 6). If presaccadic remapping does not occur (Figure 13C), the unchanged visual representation of the target is referenced to the predictively updated eye position (dashed circle in the visual field). The target therefore moves in workspace coordinates, and the arm follows the movement.

### Synchronicity of remapping and eye position updates

**Figure 14:**
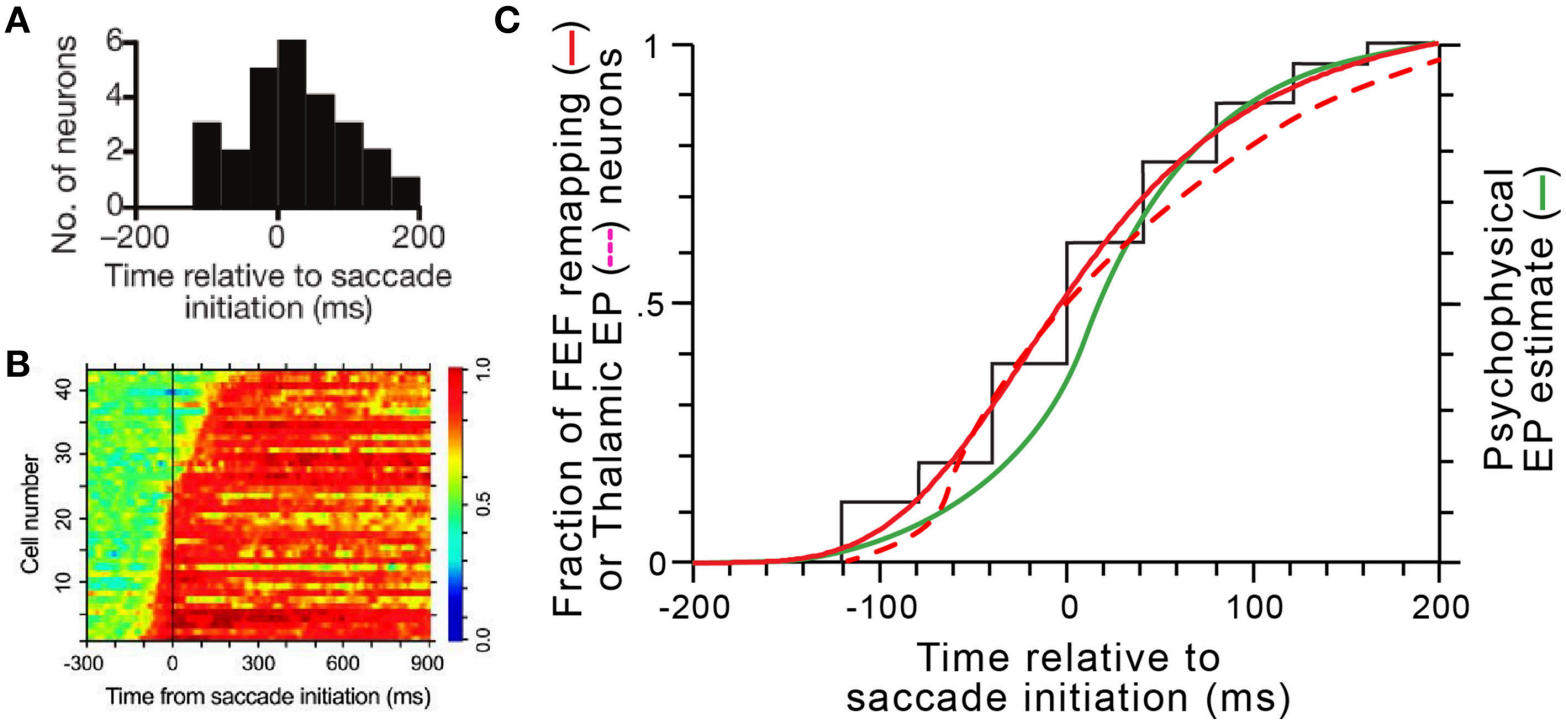
**Coordinated dynamics of remapping and eye position signals *in vivo*. (A)** The distribution of presaccadic remapping onset times for 26 FEF neurons (adapted with permission from Figure 3C of Sommer and Wurtz, 2006). **(B)** ROC analysis performed on 43 eye position related neurons in central thalamus (adapted with permission from Figure 5B of Tanaka, 2007). **(C)** Comparison of timing curves. The black “staircase” function shows the raw cumulative distribution of remapping onset times in FEF derived from panel **(A)**, and the solid red curve shows the logistic fit to that data (equation was y = [−1.08∕(1+[(*x*+200)∕204.9]^3.9^)] + 1.08. The dashed red curve shows the cumulative distribution of updates in thalamic eye position signals derived by tracing and smoothing the transitions to red across the rows of data in panel **(B)**. The green curve shows the time course of internal representations of eye position in humans calculated from a representative psychophysical experiment (modified with permission from Figure 4B of Honda, 1989).

For presaccadic remapping to counter predictive eye position signals, they must be in sync. Thus, our model explains the “when” of remapping: it needs to start at the moment eye position signals are updated. Remapping onset times have been quantified in several studies (e.g., Kusunoki and Goldberg, 2003; Sommer and Wurtz, 2006). In individual FEF neurons, onset times are consistent, but between neurons, they vary over a broad range from about 100 ms before saccade initiation to about 200 ms after, with a median almost exactly at saccade initiation (Figure 14A; median is –2 ms). Predictive eye position signals have been found, and their timings quantified, in the thalamus (Schlag-Rey and Schlag, 1984; Wyder et al., 2003; Tanaka, 2007). A summary of the timings found by Tanaka (2007) is shown in Figure 14B. He found that individual thalamic neurons update their eye position signals from about 100 ms before saccade initiation to around 300 ms after, with a median close to saccade initiation.

To compare these known, *in vivo* timings of FEF remapping (Figure 14A) and thalamic eye position updating (Figure 14B), we constructed cumulative distribution functions for both sets of data and superimposed them (solid and dashed red curves, respectively, in Figure 14C). We compared them, additionally, to internal eye position signals as derived from psychophysical studies (green curve in Figure 14C). Numerous studies in humans and monkeys have indicated that the visual system uses a “sluggish” internal eye position signal that begins to update prior to a saccade and continues to update during and after it (Honda, 1989, 1991; Dassonville et al., 1992; Schlag and Schlag-Rey, 1995; Kaiser and Lappe, 2004; Jeffries et al., 2007). Consistent with the prediction of our model, the distribution of remapping onset times overlaps almost perfectly with the distribution of neural eye position updating times and closely with the distribution of psychophysical eye position updating times (Figure 14C).

The temporal correlation between the initiation of presaccadic remapping and eye position updating (Figure 14C) is striking but circumstantial. It is possible that the FEF and thalamic signals change at the same time but are unrelated. Their functional relationship needs to be tested *in vivo*. Specifically, our model predicts that individual remapping and eye position neurons with similar time courses of activity are selectively connected. For example, in our experiment of Figure 8, we updated the eye position signal at three different times relative to saccade initiation. The model always optimized its performance by matching the timing of remapping to the timing of eye position updating. In the brain, there is a broad distribution of timings across neurons (Figures 14A,B), so the experiment of Figure 8 corresponds to isolating distinct samples from the temporal distribution of eye position signals and showing that they are temporally matched to distinct samples from the remapping distribution. This leads to the hypothesis that individual remapping neurons connect with individual eye position neurons on the basis of temporal similarity. This hypothesis is testable through at least two lines of *in vivo* experiments.

One approach could involve simultaneous recordings in FEF and thalamus. By recording from one or more FEF remapping neurons and thalamic eye position neurons, their correlated variability and synchrony could be analyzed (Smith and Sommer, 2013; Ruff and Cohen, 2014). Such analyses can be performed on short times scales for spikes (ms) and longer time scales for noise correlations (hundreds of ms) to assess the evidence for a functional connection between the neurons and the putative direction of that connection (Cohen and Kohn, 2011). We predict that the probability of a significant cross correlation or noise correlation between an FEF remapping neuron and a thalamic eye position neuron is directly related to the similarity in the neurons' signal onset times. For example, a neuron that starts remapping before the saccade is more likely to be connected (stronger correlations) to an eye position neuron that also starts updating before the saccade than to an eye position neuron that starts updating after the saccade.

Another approach could be causal. In the experiments performed by (Sommer and Wurtz, 2006), remapping neurons in the FEF were studied while the corollary discharge pathway from SC was silenced at the level of the thalamus. The strength of remapping decreased, but there was no obvious change in the timing of remapping. In analogous experiments, one could record from remapping neurons in FEF while inactivating eye position regions in the thalamus. Our model predicts that, during inactivation, the strength of remapping will not be affected but the timing of remapping will be impaired, becoming highly variable trial-by-trial or dramatically delayed overall. But before such an experiment is possible, an improved understanding of the anatomical and functional connectivity between the FEF and thalamic eye position regions is needed.

It has been emphasized previously that presaccadic remapping is better synchronized to saccade initiation than visual stimulus onset (for review see Sommer and Wurtz, 2008). If the experiments described above confirm our model's predictions, then this tenet of synchronicity with saccade initiation would need to be revised. The new conclusion would be that presaccadic remapping is synchronized, on a neuron-by-neuron basis, with predictive eye position signals. Those signals, of course, may be correlated with saccade initiation if they are produced by integration of saccade commands (see next section). But the apparent linkage of remapping to saccade initiation would be a second-order effect; the primary linkage would be with eye position signals.

### Origin of predictive eye position signals

We have focused on the eye position signals known to exist in thalamus, but where those signals come from is still unknown, and eye position signals unrelated to thalamus may be important as well. There are a number of possible origins for eye position signals. Proprioceptive information about eye position is available in somatosensory cortex (Wang et al., 2007), but its onset is postsaccadic and what it represents is unclear, as there is little evidence for sensory receptors in the primate extraocular muscles (Rao and Prevosto, 2013). In the brainstem, horizontal and vertical eye position in the orbit is sustained, respectively, by rate codes in the nucleus prepositus hypoglossi (NPH; Lopez-Barneo et al., 1982; Fukushima et al., 1992) and the interstitial nucleus of Cajal (iC; Fukushima, 1987; Crawford et al., 1991), both of which send projections to the thalamus (Kotchabhakdi et al., 1980; Kokkoroyannis et al., 1996; Prevosto et al., 2009). The NPH and iC seem to create eye position signals through integration of afferent eye velocity signals that precede saccades by only a few milliseconds. Neither structure provides an unambiguous eye position signal until after the movement. Hence, eye position signals from the NPH and iC are predictive in the sense of preceding visual afferent lags, but they are not presaccadic. Eye position signals that start well before a saccade seem more likely to derive from longer-lead activity as found in the SC and cerebral cortical areas (e.g., Wurtz et al., 2001). Presaccadic bursts of activity in SC-to-thalamus neurons are known to have the appropriate timing, with a median onset time of 85 ms before saccade initiation (Sommer and Wurtz, 2004a).

Regardless of their source, predictive eye position signals in the primate brain primarily use a rate code (higher firing rate = greater eccentricity in the orbit). In our model, we chose to simplify the representation with a two-dimensional topographic code to allow for a ready match with FEF output. A more detailed model could incorporate rate code inputs, but the end result, at the population level, would be a location in the orbit as we represented. Activity in our model's EP sheet should be thought of as the overall readout of more reductionist thalamic and/or brainstem codes.

### Potential contribution of gain fields

In this report, we have focused on explicit representations of eye position. A more implicit representation takes the form of gain fields, in which neurons' visual responses are modulated by eye position. This effect has been studied in the SC (Van Opstal et al., 1995), SEF (Schlag et al., 1992), FEF (Cassanello and Ferrera, 2007), dorsal premotor cortex (Boussaoud et al., 1998), and most extensively in the lateral intraparietal area (LIP; Andersen and Mountcastle, 1983; Andersen et al., 1985, 1993). Gain fields provide a mathematically elegant way to combine visual and positional information to solve a coordinate transformation (Zipser and Andersen, 1988; Brotchie et al., 1995; Chang et al., 2009). However, the *in vivo* temporal dynamics of gain fields are still under debate. Recent experimental work showed that gain fields in LIP are slow to update after an eye movement and do not represent eye position information until about 150 ms after a saccade (Xu et al., 2011, 2012). On the other hand, another study was able to extract predictive eye position signals using a Bayesian inference technique (Graf and Andersen, 2014). We do not rule out gain fields as a source of predictive eye position signals, but the evidence for a thalamic source seems stronger.

### Relation to previous modeling studies

Previous neural network models investigated the problem of visuospatial constancy across saccades. One approach was to use a retinocentric frame of reference and update information with a corollary discharge signal. The postulated nature of that signal and dynamics of remapping varied between models. Quaia et al. (1998) used a highly interconnected, all-to-all network to simulate visual remapping based on vector subtraction driven by a directional saccadic burst. Other such models with an instantiation of a topographically organized networks showed visual updating using the saccadic velocity commands (Droulez and Berthoz, 1991; Bozis and Moschovakis, 1998). Visual remapping can arise when the goal of a dynamic network is to maintain memory of a target across saccades (Schneegans and Schöner, 2012). In a series of models, Keith and colleagues trained a feed-forward neural network to perform remapping in single time steps and incorporated temporal dynamics by means of recurrent connections (Keith et al., 2007; Keith and Crawford, 2008). The recurrent network performed a double-step task using individual signals known to exist in the brain, such as visual error, motor bursts that begin prior to the saccade, and the saccade velocity (Keith et al., 2010). The population dynamics of the trained model depended on the updating signal. In the case of using transient visual responses to the first saccadic target, the receptive fields often jumped to the updated position. However, neurons in the hidden layer exhibited a diverse set of responses including shifts in the direction of the saccade and shifts in the opposite direction.

Neural networks also have been used to demonstrate the emergence of gain fields in hidden layer units that combine visual inputs and eye position (Zipser and Andersen, 1988; Andersen et al., 1990; Mazzoni et al., 1991). Subsequent work using multi-layered neural networks found hidden layer gain fields after training on saccade tasks that require quick spatial updating (Xing and Andersen, 2000a,b; White and Snyder, 2004). However, the velocity input was primarily used by the network and these simulated positional gain fields seem unnecessary for such tasks (White and Snyder, 2007) and, *in vivo*, their dynamics seem too slow (Xu et al., 2012).

A recent study by Wang et al. (2016) found perisaccadic expansions of receptive fields in LIP and demonstrated how a network might instantiate such an effect. There are two key differences between our model and theirs. First, their model of LIP featured lateral connections between remapping neurons in the output layer. Our model lacked lateral connections in FEF but contained fully recurrent connections in the Hidden layer that could mediate perisaccadic expansions in FEF. The second, more important difference relates to dynamics of activity in the SC layer. While Wang et al. (2016) assumed a moving hill of activity in the SC (Munoz et al., 1991; Munoz and Wurtz, 1995), we used a simpler saccadic command resembling a locus of activity on the SC topographic map. The strength, directional specificity, and functional relevance of a moving hill in the SC is debatable (Ottes et al., 1986; Anderson et al., 1998; Port et al., 2000; Soetedjo et al., 2002).

Further computational methods using radial basis-function networks have formalized the embedding and integration of multisensory information and the eye position invariant representations of targets (Deneve et al., 2001; Salinas and Sejnowski, 2001; Pouget et al., 2002). Typically, these type of networks read out noisy populations and focus on static transformations. A recurrent basis network is needed to model dynamic inputs (Deneve et al., 2007). Other models have been designed (Pola, 2004, 2007; Binda et al., 2009), for review, see (Hamker et al., 2011), to examine perceptual errors in spatial localization around the time of the saccade (Matin and Pearce, 1965; Honda, 1989, 1991; Dassonville et al., 1992; Schlag and Schlag-Rey, 1995). The key feature of such models is an eye position signal that is predictive but sluggish, continuing through the movement. Finally, several studies show that such a perceptual mislocalization can arises from a Bayes-optimal transsaccadic integration (Niemeier et al., 2003; Teichert et al., 2010).

In sum, previous modeling efforts used myriad approaches to understanding visual stability across saccades. Most of them were abstract representations of the primate brain, while those that were more neuromorphic (e.g., Quaia et al., 1998) have not been tested in large-scale simulations or updated to incorporate new *in vivo* data. The main contribution of our approach was to use a less abstract, more biologically inspired architecture that took into account the latest findings on oculomotor circuits. Our hierarchical, sheet-based model, due to its close structural correspondence with the primate brain, is well-suited for informing future neurophysiological studies and easily updatable in response to new data from such studies.

### Continuously present vs. flashed visual stimuli

Some of the prior modeling studies of spatial updating focused on briefly flashed probes and the remapping of visual memory (White and Snyder, 2004; Keith et al., 2010; Schneegans and Schöner, 2012). A subset of this class of models (for review, see Hamker et al., 2008; Ziesche and Hamker, 2011, 2014) aimed to explain an intriguing illusion, transsaccadic mislocalization, that can accompany the viewing of brief flashes (Dassonville et al., 1992; Kaiser and Lappe, 2004; Jeffries et al., 2007). The use of brief flashes in modeling has yielded many insights into possible underlying mechanisms of spatial updating, and it mimics a large body of laboratory work, including studies of presaccadic remapping (e.g., Sommer and Wurtz, 2006; Shin and Sommer, 2012). Neurons exhibit presaccadic remapping for more persistent stimuli as well, however (Duhamel et al., 1992; Umeno and Goldberg, 1997; Kusunoki and Goldberg, 2003), including continuously present stimuli (Mirpour and Bisley, 2012).

We kept our paradigm simple, using continuously present stimuli rather than stimuli flashed prior to a saccade. The latter approach would require an unnecessary level of complexity caused by memory responses. If a computational network such as ours is tasked with holding the memory of a visual probe, the weights in the recurrent connections adapt to maintain activity at that spatial location, and the influence of the input neurons are nulled after the first time point (Xing and Andersen, 2000b). We aimed to study the separate influences of visual and CD inputs through the course of remapping by systematically training them and then selectively lesioning them (White and Snyder, 2004, 2007). Using a persistent visual probe obviated the need for recurrent connections to multiplex the learning of memory with the learning of optimal temporal relationships between inputs. Had we designed the network to accommodate flashed visual inputs, memory mechanisms in the recurrent connections could have obscured the deficits we observed. More generally, our motivation was to study everyday visuomotor behavior. From an ecological perspective, it is rare that a behaviorally-relevant stimulus appears for only tens of milliseconds, just before a saccade.

### Limitations and future directions

In the present study, we briefly touched upon the values of the weights between connections. We observed that, at the Hidden layer, weights stemming from the Retina layer were stronger than those stemming from the SC layer. Additional work is needed to determine the detailed contributions of each layer as well as the recurrent connections. Manipulating and understanding the connectivity in more detail may lead to a better understanding of the temporal dynamics of presaccadic remapping.

We made no restrictions on what the weights of the connections could be. A single neuron could send excitatory projections to one set of neurons and inhibitory projections to another. Previous work on the microcircuitry within the FEF has revealed differing roles of excitatory and inhibitory neurons in presaccadic remapping (Shin and Sommer, 2012). One could constrain the weights of a neuron as per Dale's principle such that each one has either an excitatory or inhibitory effect. Investigation and selective manipulation of subsets of neurons from that point would yield insights in the roles of each sub-group on the process of remapping, and could lead to a more neuromorphic model even at the level of microcircuitry between and within FEF layers.

We imposed simple spatiotemporal dynamics on activity in the SC and EP layers. The biological signals, however, may be more nuanced, especially in the SC (Munoz and Wurtz, 1995; Anderson et al., 1998; Port et al., 2000; Soetedjo et al., 2002). Future versions of the model could vary the spatiotemporal dynamics of activity in these two layers to assess the consequences for receptive field remapping and useful stability.

Finally, every connection within the network was “all-to-all.” Visual neurons have specific anatomical connectivity that constrains their receptive field, however, so an all-to-all connection from the Retina to the FEF is probably not biologically accurate. Further, our knowledge of the topography with which SC projects to MD and so to the FEF is limited (Sommer and Wurtz, 2004a). A useful next step would be to constrain the topographic distribution of projections between the Retina, SC, Hidden, and FEF layers, and then train the network with the same criteria used in this report. Such an investigation would move the model toward better biological plausibility, although we see no reason to suspect that it would result in different overall conclusions.

## Conclusion

We developed a computational model to probe the underlying mechanisms of visual stability. Our approach was to study visually-guided action. By enforcing that our model guided actions that require visual stability, we trained it to achieve a state that, if observed in a human, would imply perceptual stability. During model training, performance improved in tight correlation with the emergence of presaccadic remapping in a simulated FEF. The remapping depended on an intact corollary discharge pathway as found *in vivo*. Spatially, the remapping took the form of decreased activity at the Receptive Field coupled with increased activity at the Future Field, as found in the biological FEF. Temporally, the onset of presaccadic remapping was synchronized to the predictive updating of eye position signals, a relationship that was not appreciated previously. The model provided a novel explanation for the variability of remapping onset times measured *in vivo*, and it led to a hypothesis, testable by multiple physiological approaches, that predictive eye position entrains presaccadic remapping.

Our model focused on useful visual stability, the ability to interact with the world while moving the eyes. We did not directly model perceptual visual stability, the experience of an unperturbed visual scene while moving the eyes. It is clear why useful visual stability would evolve. Natural selection would favor neural circuits that achieve coordinate transformations, and thus accurate multi-segmented actions, as fast as possible. Our model identified presaccadic remapping as one mechanism that contributes to quick, even predictive, coordinate transformations. It is plausible that once presaccadic remapping evolved for useful visual stability, it was exploited for other potential ends such as perceptual visual stability (e.g., Gould and Lewontin, 1979).

## Author contributions

HR and MS conceived the work and wrote the manuscript. HR, JS, FS, JV, and KR wrote the code, performed the experiments, and analyzed the data.

### Conflict of interest statement

The authors declare that the research was conducted in the absence of any commercial or financial relationships that could be construed as a potential conflict of interest.

## References

[B1] Andersen R. A. Bracewell R. M. Barash S. Gnadt J. W. Fogassi L. (1990). Eye position effects on visual, memory, and saccade-related activity in areas LIP and 7a of macaque. J. Neurosci. 10, 1176–1196 2329374 10.1523/JNEUROSCI.10-04-01176.1990 PMC6570201

[B2] Andersen R. A. Essick G. K. Siegel R. M. (1985). Encoding of spatial location by posterior parietal neurons. Science 230, 456–458. 10.1126/science.4048942 4048942

[B3] Andersen R. A. Mountcastle V. B. (1983). The influence of the angle of gaze upon the excitability of the light-sensitive neurons of the posterior parietal cortex. J. Neurosci. 3, 532–548. 6827308 10.1523/JNEUROSCI.03-03-00532.1983 PMC6564545

[B4] Andersen R. A. Snyder L. H. Li C. S. Stricanne B. (1993). Coordinate transformations in the representation of spatial information. Curr. Opin. Neurobiol. 3, 171–176. 10.1016/0959-4388(93)90206-E 8513228

[B5] Anderson R. W. Keller E. L. Gandhi N. J. Das S. (1998). Two-dimensional saccade-related population activity in superior colliculus in monkey. J. Neurophysiol. 80, 798–817. 9705470 10.1152/jn.1998.80.2.798

[B6] Bednar J. A. Choe Y. De Paula J. Miikkulainen R. Provost J. Tversky T. (2004). Modeling cortical maps with Topographica. Neurocomputing 58, 1129–1135. 10.1016/j.neucom.2004.01.177

[B7] Bednar J. A. Miikkulainen R. (2003). Self-organization of spatiotemporal receptive fields and laterally connected direction and orientation maps. Neurocomputing 52, 473–480. 10.1016/S0925-2312(02)00735-X

[B8] Bednar J. A. (2008). Understanding neural maps with topographica. Brains Minds Media 3:bmm1402 Available online at: http://www.brains-minds-media.org/

[B9] Bednar J. A. (2009). Topographica: building and analyzing map-level simulations from Python, C/C++, MATLAB, NEST, or NEURON components. Front. Neuroinform. 3:8. 10.3389/neuro.11.008.2009 19352443 PMC2666198

[B10] Bellebaum C. Daum I. Koch B. Schwarz M. Hoffmann K. P. (2005). The role of the human thalamus in processing corollary discharge. Brain 128, 1139–1154. 10.1093/brain/awh474 15758033

[B11] Binda P. Cicchini G. M. Burr D. C. Morrone M. C. (2009). Spatiotemporal distortions of visual perception at the time of saccades. J. Neurosci. 29, 13147–13157. 10.1523/JNEUROSCI.3723-09.2009 19846702 PMC6665185

[B12] Boussaoud D. Barth T. M. Wise S. P. (1993). Effects of gaze on apparent visual responses of frontal cortex neurons. Exp. Brain Res. 93, 423–434. 10.1007/BF00229358 8519333

[B13] Boussaoud D. Jouffrais C. Bremmer F. (1998). Eye position effects on the neuronal activity of dorsal premotor cortex in the macaque monkey. J. Neurophysiol. 80, 1132–1150. 9744928 10.1152/jn.1998.80.3.1132

[B14] Bozis A. Moschovakis A. K. (1998). Neural network simulations of the primate oculomotor system III. A one-dimensional, one-directional model of the superior colliculus. Biol. Cybern. 79, 215–230. 10.1007/s004220050472 9810679

[B15] Brotchie P. R. Andersen R. A. Snyder L. H. Goodman S. J. (1995). Head position signals used by parietal neurons to encode locations of visual stimuli. Nature 375, 232–235. 10.1038/375232a0 7746323

[B16] Cassanello C. R. Ferrera V. P. (2007). Computing vector differences using a gain field-like mechanism in monkey frontal eye field. J. Physiol. 582, 647–664. 10.1113/jphysiol.2007.128801 17510192 PMC2075335

[B17] Cavanaugh J. Berman R. A. Joiner W. M. Wurtz R. H. (2016). Saccadic corollary discharge underlies stable visual perception. J. Neurosci. 36, 31–42. 10.1523/JNEUROSCI.2054-15.2016 26740647 PMC4701964

[B18] Chang S. W. Papadimitriou C. Snyder L. H. (2009). Using a compound gain field to compute a reach plan. Neuron 64, 744–755. 10.1016/j.neuron.2009.11.005 20005829 PMC2811884

[B19] Cohen M. R. Kohn A. (2011). Measuring and interpreting neuronal correlations. Nat. Neurosci. 14, 811–819. 10.1038/nn.2842 21709677 PMC3586814

[B20] Crapse T. B. Sommer M. A. (2009). Frontal eye field neurons with spatial representations predicted by their subcortical input. J. Neurosci. 29, 5308–5318. 10.1523/JNEUROSCI.4906-08.2009 19386927 PMC2700632

[B21] Crapse T. B. Sommer M. A. (2012). Frontal eye field neurons assess visual stability across saccades. J. Neurosci. 32, 2835–2845. 10.1523/JNEUROSCI.1320-11.2012 22357866 PMC3566788

[B22] Crawford J. D. Cadera W. Vilis T. (1991). Generation of torsional and vertical eye position signals by the interstitial nucleus of Cajal. Science 252, 1551–1553. 10.1126/science.2047862 2047862

[B23] Dassonville P. Schlag J. Schlag-Rey M. (1992). Oculomotor localization relies on a damped representation of saccadic eye displacement in human and nonhuman primates. Vis. Neurosci. 9, 261–269. 10.1017/S0952523800010671 1390386

[B24] Deneve S. Duhamel J. R. Pouget A. (2007). Optimal sensorimotor integration in recurrent cortical networks: a neural implementation of Kalman filters. J. Neurosci. 27, 5744–5756. 10.1523/JNEUROSCI.3985-06.2007 17522318 PMC6672763

[B25] Deneve S. Latham P. E. Pouget A. (2001). Efficient computation and cue integration with noisy population codes. Nat. Neurosci. 4, 826–831. 10.1038/90541 11477429

[B26] Droulez J. Berthoz A. (1991). A neural network model of sensoritopic maps with predictive short-term memory properties. Proc. Natl. Acad. Sci. U.S.A. 88, 9653–9657. 10.1073/pnas.88.21.9653 1946381 PMC52776

[B27] Duhamel J. R. Colby C. L. Goldberg M. E. (1992). The updating of the representation of visual space in parietal cortex by intended eye movements. Science 255, 90–92. 10.1126/science.1553535 1553535

[B28] Fuchs A. F. Kaneko C. R. S. Scudder C. A. (1985). Brainstem control of saccadic eye movements. Annu. Rev. Neurosci. 8, 307–337. 10.1146/annurev.ne.08.030185.001515 3920944

[B29] Fukushima K. (1987). The interstitial nucleus of Cajal and its role in the control of movements of head and eyes. Prog. Neurobiol. 29, 107–192. 10.1016/0301-0082(87)90016-5 3108957

[B30] Fukushima K. Kaneko C. R. Fuchs A. F. (1992). The neuronal substrate of integration in the oculomotor system. Prog. Neurobiol. 39, 609–639. 10.1016/0301-0082(92)90016-8 1410443

[B31] Gould S. J. Lewontin R. C. (1979). The spandrels of San Marco and the Panglossian paradigm: a critique of the adaptationist programme. Proc. R. Soc. Lond. B Biol. Sci. 205, 581–598. 10.1098/rspb.1979.0086 42062

[B32] Graf A. B. Andersen R. A. (2014). Inferring eye position from populations of lateral intraparietal neurons. Elife 3:e02813. 10.7554/eLife.02813 24844707 PMC4021542

[B33] Hamker F. H. Zirnsak M. Calow D. Lappe M. (2008). The peri-saccadic perception of objects and space. PLoS Comput. Biol. 4:e31. 10.1371/journal.pcbi.0040031 18282086 PMC2242822

[B34] Hamker F. H. Zirnsak M. Ziesche A. Lappe M. (2011). Computational models of spatial updating in peri-saccadic perception. Philos. Trans. R. Soc. B Biol. Sci. 366, 554–571. 10.1098/rstb.2010.0229 21242143 PMC3030832

[B35] Holst E. V. Mittelstaedt H. (1971). The principle of reafference: interactions between the central nervous system and the peripheral organs, Perceptual Processing: Stimulus Equivalence and Pattern Recognition, ed Dodwell P. C. (New York, NY: Appleton-Century-Crofts, Meredith Corporation), 41–71.

[B36] Honda H. (1989). Perceptual localization of visual stimuli flashed during saccades. Percept. Psychophys. 45, 162–174. 10.3758/BF03208051 2928078

[B37] Honda H. (1991). The time courses of visual mislocalization and of extraretinal eye position signals at the time of vertical saccades. Vision Res. 31, 1915–1921. 10.1016/0042-6989(91)90186-9 1771775

[B38] Jeffries S. M. Kusunoki M. Bisley J. W. Cohen I. S. Goldberg M. E. (2007). Rhesus monkeys mislocalize saccade targets flashed for 100 ms around the time of a saccade. Vision Res. 47, 1924–1934. 10.1016/j.visres.2007.02.021 17499832 PMC2367055

[B39] Kaiser M. Lappe M. (2004). Perisaccadic mislocalization orthogonal to saccade direction. Neuron 41, 293–300. 10.1016/S0896-6273(03)00849-3 14741109

[B40] Keith G. P. Blohm G. Crawford J. D. (2010). Influence of saccade efference copy on the spatiotemporal properties of remapping: a neural network study. J. Neurophysiol. 103, 117–139. 10.1152/jn.91191.2008 19846615

[B41] Keith G. P. Crawford J. D. (2008). Saccade-related remapping of target representations between topographic maps: a neural network study. J. Comput. Neurosci. 24, 157–178. 10.1007/s10827-007-0046-6 17636448

[B42] Keith G. P. Smith M. A. Crawford J. D. (2007). Functional organization within a neural network trained to update target representations across 3-D saccades. J. Comput. Neurosci. 22, 191–209. 10.1007/s10827-006-0007-5 17120151

[B43] Kokkoroyannis T. Scudder C. A. Balaban C. D. Highstein S. M. Moschovakis A. K. (1996). Anatomy and physiology of the primate interstitial nucleus of Cajal I. Efferent projections. J. Neurophysiol. 75, 725–739. 8714648 10.1152/jn.1996.75.2.725

[B44] Kotchabhakdi N. Rinvik E. Yingchareon K. Walberg F. (1980). Afferent projections to the thalamus from the perihypoglossal nuclei. Brain Res. 187, 457–461. 10.1016/0006-8993(80)90215-2 7370739

[B45] Kusunoki M. Goldberg M. E. (2003). The time course of perisaccadic receptive field shifts in the lateral intraparietal area of the monkey. J. Neurophysiol. 89, 1519–1527. 10.1152/jn.00519.2002 12612015

[B46] Lopez-Barneo J. Darlot C. Berthoz A. Baker R. (1982). Neuronal activity in prepositus nucleus correlated with eye movement in the alert cat. J. Neurophysiol. 47, 329–352. 7062103 10.1152/jn.1982.47.2.329

[B47] Lynch J. C. Hoover J. E. Strick P. L. (1994). Input to the primate frontal eye field from the substantia nigra, superior colliculus, and dentate nucleus demonstrated by transneuronal transport. Exp. Brain Res. 100, 181–186. 10.1007/BF00227293 7813649

[B48] Marino A. C. Mazer J. A. (2016). Perisaccadic updating of visual representations and attentional states: linking behavior and neurophysiology. Front. Syst. Neurosci. 10:3. 10.3389/fnsys.2016.00003 26903820 PMC4743436

[B49] Matin L. Pearce D. G. (1965). Visual perception of direction for stimuli flashed during voluntary saccadic eye movements. Science 148, 1485–1488. 10.1126/science.148.3676.1485 17738160

[B50] Mayo J. P. DiTomasso A. R. Sommer M. A. Smith M. A. (2015). Dynamics of visual receptive fields in the macaque frontal eye field. J. Neurophysiol. 114, 3201–3210. 10.1152/jn.00746.2015 26378208 PMC4686296

[B51] Mayo J. P. Morrison R. M. Smith M. A. (2016). A probabilistic approach to receptive field mapping in the frontal eye fields. Front. Syst. Neurosci. 10:25. 10.3389/fnsys.2016.00025 27047352 PMC4796031

[B52] Mazzoni P. Andersen R. A. Jordan M. I. (1991). A more biologically plausible learning rule for neural networks. Proc. Natl. Acad. Sci. U.S.A. 88, 4433–4437. 10.1073/pnas.88.10.4433 1903542 PMC51674

[B53] Melcher D. Colby C. L. (2008). Trans-saccadic perception. Trends Cogn. Sci. 12, 466–473. 10.1016/j.tics.2008.09.003 18951831

[B54] Mirpour K. Bisley J. W. (2012). Anticipatory remapping of attentional priority across the entire visual field. J. Neurosci. 32, 16449–16457. 10.1523/JNEUROSCI.2008-12.2012 23152627 PMC3508767

[B55] Moschovakis A. K. (1996). The superior colliculus and eye movement control. Curr. Opin. Neurobiol. 6, 811–816. 10.1016/S0959-4388(96)80032-8 9000018

[B56] Munoz D. P. Guitton D. Pelisson D. (1991). Control of orienting gaze shifts by the tectoreticulospinal system in the head-free cat. III. Spatiotemporal characteristics of phasic motor discharges. J. Neurophysiol. 66, 1642–1666. 1765799 10.1152/jn.1991.66.5.1642

[B57] Munoz D. P. Wurtz R. H. (1995). Saccade-related activity in monkey superior colliculus I I. Spread of activity during saccades. J. Neurophysiol. 73, 2334–2348. 7666142 10.1152/jn.1995.73.6.2334

[B58] Nakamura K. Colby C. L. (2002). Updating of the visual representation in monkey striate and extrastriate cortex during saccades. Proc. Natl. Acad. Sci. U.S.A. 99, 4026–4031. 10.1073/pnas.052379899 11904446 PMC122642

[B59] Neupane S. Guitton D. Pack C. C. (2016). Two distinct types of remapping in primate cortical area V4. Nat. Commun. 7:10402. 10.1038/ncomms10402 26832423 PMC4740356

[B60] Neupane S. Guitton D. Pack C. C. (in press). Dissociation of forward convergent remapping in primate visual cortex. Curr. Biol. 26, R1–R3. 27326707 10.1016/j.cub.2016.04.050

[B61] Niemeier M. Crawford J. D. Tweed D. B. (2003). Optimal transsaccadic integration explains distorted spatial perception. Nature 422, 76–80. 10.1038/nature01439 12621435

[B62] Nowak L. G. Munk M. H. J. Girard P. Bullier J. (1995). Visual latencies in areas V1 and V2 of the macaque monkey. Vis. Neurosci. 12, 371–384. 7786857 10.1017/s095252380000804x

[B63] Ostendorf F. Liebermann D. Ploner C. J. (2010). Human thalamus contributes to perceptual stability across eye movements. Proc. Natl. Acad. Sci. U.S.A. 107, 1229–1234. 10.1073/pnas.0910742107 20080657 PMC2824294

[B64] Ottes F. P. Van Gisbergen J. A. Eggermont J. J. (1986). Visuomotor fields of the superior colliculus: a quantitative model. Vision Res. 26, 857–873. 10.1016/0042-6989(86)90144-6 3750869

[B65] Pola J. (2004). Models of the mechanism underlying perceived location of a perisaccadic flash. Vision Res. 44, 2799–2813. 10.1016/j.visres.2004.06.008 15342224

[B66] Pola J. (2007). A model of the mechanism for the perceived location of a single flash and two successive flashes presented around the time of a saccade. Vision Res. 47, 2798–2813. 10.1016/j.visres.2007.07.005 17767942

[B67] Port N. L. Sommer M. A. Wurtz R. H. (2000). Multielectrode evidence for spreading activity across the superior colliculus movement map. J. Neurophysiol. 84, 344–357. Available online at: http://jn.physiology.org/content/84/1/344.long 10899209 10.1152/jn.2000.84.1.344

[B68] Pouget A. Deneve S. Duhamel J. R. (2002). A computational perspective on the neural basis of multisensory spatial representations. Nat. Rev. Neurosci. 3, 741–747. 10.1038/nrn914 12209122

[B69] Prevosto V. Graf W. Ugolini G. (2009). Posterior parietal cortex areas MIP and LIPv receive eye position and velocity inputs via ascending preposito-thalamo-cortical pathways. Eur. J. Neurosci. 30, 1151–1161. 10.1111/j.1460-9568.2009.06885.x 19735295

[B70] Quaia C. Optican L. M. Goldberg M. E. (1998). The maintenance of spatial accuracy by the perisaccadic remapping of visual receptive fields. Neural Netw. 11, 1229–1240. 10.1016/S0893-6080(98)00069-0 12662746

[B71] Rao H. M. Prevosto V. (2013). Proprioceptive eye position signals are still missing a sensory receptor. J. Neurosci. 33, 10585–10587. 10.1523/JNEUROSCI.1594-13.2013 23804081 PMC6618496

[B72] Ross J. Morrone M. C. Burr D. C. (1997). Compression of visual space before saccades. Nature 386, 598–601. 10.1038/386598a0 9121581

[B73] Ross J. Morrone M. C. Goldberg M. E. Burr D. C. (2001). Changes in visual perception at the time of saccades. Trends Neurosci. 24, 113–121. 10.1016/S0166-2236(00)01685-4 11164942

[B74] Ruff D. A. Cohen M. R. (2014). Attention can either increase or decrease spike count correlations in visual cortex. Nat. Neurosci. 17, 1591–1597. 10.1038/nn.3835 25306550 PMC4446056

[B75] Rumelhart D. E. Hinton G. E. Williams R. J. (1988). Learning representations by back-propagating errors. Cogn. Model. 5, 1.

[B76] Salinas E. Sejnowski T. J. (2001). Book review: gain modulation in the central nervous system: where behavior, neurophysiology, and computation meet. Neuroscientist 7, 430–440. 10.1177/107385840100700512 11597102 PMC2887717

[B77] Schlag J. Schlag-Rey M. (1995). Illusory localization of stimuli flashed in the dark before saccades. Vision Res. 35, 2347–2357. 10.1016/0042-6989(95)00021-Q 7571470

[B78] Schlag J. Schlag-Rey M. Pigarev I. (1992). Supplementary eye field: influence of eye position on neural signals of fixation. Exp. Brain Res. 90, 302–306. 10.1007/BF00227242 1397144

[B79] Schlag-Rey M. Schlag J. (1984). Visuomotor functions of central thalamus in monkey. I. Unit activity related to spontaneous eye movements. J. Neurophysiol. 51, 1149–1174. 6737026 10.1152/jn.1984.51.6.1149

[B80] Schneegans S. Schöner G. (2012). A neural mechanism for coordinate transformation predicts pre-saccadic remapping. Biol. Cybern. 106, 89–109. 10.1007/s00422-012-0484-8 22481644

[B81] Shin S. Sommer M. A. (2012). Division of labor in frontal eye field neurons during presaccadic remapping of visual receptive fields. J. Neurophysiol. 108, 2144–2159. 10.1152/jn.00204.2012 22815407 PMC3545025

[B82] Smith M. A. Sommer M. A. (2013). Spatial and temporal scales of neuronal correlation in visual area V4. J. Neurosci. 33, 5422–5432. 10.1523/JNEUROSCI.4782-12.2013 23516307 PMC3712790

[B83] Soetedjo R. Kaneko C. R. Fuchs A. F. (2002). Evidence against a moving hill in the superior colliculus during saccadic eye movements in the monkey. J. Neurophysiol. 87, 2778–2789. 10.1152/jn.00974.2001 12037180

[B84] Sommer M. A. Wurtz R. H. (2002). A pathway in primate brain for internal monitoring of movements. Science 296, 1480–1482. 10.1126/science.1069590 12029137

[B85] Sommer M. A. Wurtz R. H. (2004a). What the brain stem tells the frontal cortex. *I*. Oculomotor signals sent from superior colliculus to frontal eye field via mediodorsal thalamus. J. Neurophysiol. 91, 1381–1402. 10.1152/jn.00738.2003 14573558

[B86] Sommer M. A. Wurtz R. H. (2004b). What the brain stem tells the frontal cortex. II. Role of the SC-MD-FEF pathway in corollary discharge. J. Neurophysiol. 91, 1403–1423. 10.1152/jn.00740.2003 14573557

[B87] Sommer M. A. Wurtz R. H. (2006). Influence of the thalamus on spatial visual processing in frontal cortex. Nature 444, 374–377. 10.1038/nature05279 17093408

[B88] Sommer M. A. Wurtz R. H. (2008). Brain circuits for the internal monitoring of movements. Annu. Rev. Neurosci. 31, 317–338. 10.1146/annurev.neuro.31.060407.125627 18558858 PMC2813694

[B89] Sparks D. L. (1986). Translation of sensory signals into commands for control of saccadic eye movements: role of primate superior colliculus. Physiol. Rev. 66, 118–171. 3511480 10.1152/physrev.1986.66.1.118

[B90] Sperry R. W. (1950). Neural basis of the spontaneous optokinetic response produced by visual inversion. J. Comp. Physiol. Psychol. 43, 482. 10.1037/h0055479 14794830

[B91] Tanaka M. (2006). Inactivation of the central thalamus delays self-timed saccades. Nat. Neurosci. 9, 20–22. 10.1038/nn1617 16341209

[B92] Tanaka M. (2007). Spatiotemporal properties of eye position signals in the primate central thalamus. Cereb. Cortex 17, 1504–1515. 10.1093/cercor/bhl061 16923780

[B93] Teichert T. Klingenhoefer S. Wachtler T. Bremmer F. (2010). Perisaccadic mislocalization as optimal percept. J. Vis. 10, 19. 10.1167/10.8.19 20884594

[B94] Tolias A. S. Moore T. Smirnakis S. M. Tehovnik E. J. Siapas A. G. Schiller P. H. (2001). Eye movements modulate visual receptive fields of V4 neurons. Neuron 29, 757–767. 10.1016/S0896-6273(01)00250-1 11301034

[B95] Umeno M. M. Goldberg M. E. (1997). Spatial processing in the monkey frontal eye field. I. Predictive visual responses. J. Neurophysiol. 78, 1373–1383. 9310428 10.1152/jn.1997.78.3.1373

[B96] Van Opstal A. J. Hepp K. Suzuki Y. Henn V. (1995). Influence of eye position on activity in monkey superior colliculus. J. Neurophysiol. 74, 1593–1610. 8989396 10.1152/jn.1995.74.4.1593

[B97] von Helmholtz H. (2000). Helmholtz's Treatise on Physiological Optics, Vol. 3. Transl. by J. P. C. Southall. Bristol: Thoemmes Press.

[B98] Walker M. F. Fitzgibbon E. J. Goldberg M. E. (1995). Neurons in the monkey superior colliculus predict the visual result of impending saccadic eye movements. J. Neurophysiol. 73, 1988–2003. 7623096 10.1152/jn.1995.73.5.1988

[B99] Wang X. Fung C. A. Guan S. Wu S. Goldberg M. E. Zhang M. (2016). Perisaccadic receptive field expansion in the lateral intraparietal area. Neuron 90, 400–409. 10.1016/j.neuron.2016.02.035 27041502 PMC7035788

[B100] Wang X. Zhang M. Cohen I. S. Goldberg M. E. (2007). The proprioceptive representation of eye position in monkey primary somatosensory cortex. Nat. Neurosci. 10, 640–646. 10.1038/nn1878 17396123

[B101] White R. L. Snyder L. H. (2004). A neural network model of flexible spatial updating. J. Neurophysiol. 91, 1608–1619. 10.1152/jn.00277.2003 14668290

[B102] White R. L. Snyder L. H. (2007). Spatial constancy and the brain: insights from neural networks. Philos. Trans. R. Soc. B Biol. Sci. 362, 375–382. 10.1098/rstb.2006.1965 17255021 PMC2323556

[B103] Williams R. J. Zipser D. (1995). Gradient-based learning algorithms for recurrent networks and their computational complexity, in Backpropagation: Theory, Architectures, and Applications, eds Chauvin Y. Rumelhart D. E. (Hillsdale, NJ: Lawrence Erlbaum Associates, Publishers), 433–486.

[B104] Wurtz R. H. Joiner W. M. Berman R. A. (2011). Neuronal mechanisms for visual stability: progress and problems. Philos. Trans. R. Soc. B Biol. Sci. 366, 492–503. 10.1098/rstb.2010.0186 21242138 PMC3030829

[B105] Wurtz R. H. Sommer M. A. Paré M. Ferraina S. (2001). Signal transformations from cerebral cortex to superior colliculus for the generation of saccades. Vision Res. 41, 3399–3412. 10.1016/S0042-6989(01)00066-9 11718782

[B106] Wurtz R. H. (2008). Neuronal mechanisms of visual stability. Vision Res. 48, 2070–2089. 10.1016/j.visres.2008.03.021 18513781 PMC2556215

[B107] Wyder M. T. Massoglia D. P. Stanford T. R. (2003). Quantitative assessment of the timing and tuning of visual-related, saccade-related, and delay period activity in primate central thalamus. J. Neurophysiol. 90, 2029–2052. 10.1152/jn.00064.2003 12724361

[B108] Xing J. Andersen R. (2000a). Models of the posterior parietal cortex which perform multimodal integration and represent space in several coordinate frames. J. Cogn. Neurosci. 12, 601–614. 10.1162/089892900562363 10936913

[B109] Xing J. Andersen R. A. (2000b). Memory activity of LIP neurons for sequential eye movements simulated with neural networks. J. Neurophysiol. 84, 651–665. Available online at: http://jn.physiology.org/content/84/2/651.long 10938293 10.1152/jn.2000.84.2.651

[B110] Xu B. Y. Karachi C. Goldberg M. E. (2012). The postsaccadic unreliability of gain fields renders it unlikely that the motor system can use them to calculate target position in space. Neuron 76, 1201–1209. 10.1016/j.neuron.2012.10.034 23259954 PMC3673542

[B111] Xu Y. Wang X. Peck C. Goldberg M. E. (2011). The time course of the tonic oculomotor proprioceptive signal in area 3a of somatosensory cortex. J. Neurophysiol. 106, 71–77. 10.1152/jn.00668.2010 21346201 PMC3129727

[B112] Ziesche A. Hamker F. H. (2011). A computational model for the influence of corollary discharge and proprioception on the perisaccadic mislocalization of briefly presented stimuli in complete darkness. J. Neurosci. 31, 17392–17405. 10.1523/JNEUROSCI.3407-11.2011 22131401 PMC6623809

[B113] Ziesche A. Hamker F. H. (2014). Brain circuits underlying visual stability across eye movements—converging evidence for a neuro-computational model of area LIP. Front. Comput. Neurosci. 8:25. 10.3389/fncom.2014.00025 24653691 PMC3949326

[B114] Zipser D. Andersen R. A. (1988). A back-propagation programmed network that simulates response properties of a subset of posterior parietal neurons. Nature 331, 679–684. 10.1038/331679a0 3344044

[B115] Zirnsak M. Steinmetz N. A. Noudoost B. Xu K. Z. Moore T. (2014). Visual space is compressed in prefrontal cortex before eye movements. Nature 507, 504–507. 10.1038/nature13149 24670771 PMC4064801

